# Exploring the association between rosacea and acne by integrated bioinformatics analysis

**DOI:** 10.1038/s41598-024-53453-x

**Published:** 2024-02-06

**Authors:** Jingchen Liang, Ying Chen, Zihao Wang, Yawen Wang, Shengzhi Mu, Dewu Zhang, Zhao Wang, Weihui Zeng

**Affiliations:** 1 Department of Dermatology, The Second Affiliated Hospital of Xi’an Jiaotong University, Xi’an, China; 2 Department of Cardiology, The First Affiliated Hospital of Xi’an Jiaotong University, Xi’an, China; 3 Department of Burn and Plastic Surgery, Shaanxi Provincial People’s Hospital, Xi’an, China

**Keywords:** Mechanisms of disease, Cell signalling

## Abstract

Clinically, rosacea occurs frequently in acne patients, which hints the existence of shared signals. However, the connection between the pathophysiology of rosacea and acne are not yet fully understood. This study aims to unveil molecular mechanism in the pathogenesis of rosacea and acne. We identified differentially expressed genes (DEGs) by limma and weighted gene co-expression network analysis and screened hub genes by constructing a protein–protein interaction network. The hub genes were verified in different datasets. Then, we performed a correlation analysis between the hub genes and the pathways. Finally, we predicted and verified transcription factors of hub genes, performed the immune cell infiltration analysis using CIBERSORT, and calculated the correlation between hub genes and immune cells. A total of 169 common DEGs were identified, which were mainly enriched in immune-related pathways. Finally, hub genes were identified as IL1B, PTPRC, CXCL8, MMP9, CCL4, CXCL10, CD163, CCR5, CXCR4, and TLR8. 9 transcription factors that regulated the expression of hub genes were identified. The infiltration of γδT cells was significantly increased in rosacea and acne lesions and positively linked with almost all hub genes. These identified hub genes and immune cells may play a crucial role in the development of rosacea and acne.

## Introduction

Rosacea is a chronic inflammatory disease that typically affects women over 30. Clinically, rosacea can be divided into four subtypes: erythematotelangiectatic rosacea (ETR), papulopustular rosacea (PPR), phymatous rosacea (PHR), and ocular rosacea (OR). According to the global ROSacea COnsensus 2019 panel, the major features of rosacea include: (a) Temporary increase in centrofacial redness, which may include sensations of warmth, heat, burning and/or pain; (b) Red papules and pustules, usually in the centrofacial area. Some may be larger and deeper; (c) Visible vessels in the centrofacial region but not only in the alar area^1^. Rosacea can be induced by external factors such as sun exposure, heat and cold, hot drinks, and spicy food, but its specific etiology remains unknown^2^.

Acne vulgaris (also known as acne) is also a chronic inflammatory disease that mostly affects the face, neck, chest, and back. Acne characterized by inflammatory papules, pustules, nodules, cysts, etc. It may be caused by androgen-induced increased sebum production, altered keratinization, inflammation, and bacterial colonization of hair follicles by *Propionibacterium acnes* (*P. acnes*)^3^. Although some predisposing factors have been identified, exactly what triggers acne and how treatment affects the course of the disease remain unclear^3^. Due to the clinical characteristics of these two diseases, they frequently have substantial negative impacts on mood and quality of life of patients and, in extreme circumstances, can induce anxiety and depression^4^.

Clinically, people with rosacea are often combined with other skin diseases, such as acne, folliculitis, seborrheic dermatitis, and hormone-dependent dermatitis, especially acne. And we also frequently see patients who develop from acne to rosacea. According to Chen’s study, ETR is the most common rosacea type in 563 female acne patients combined with rosacea^5^. Due to the fact that acne also includes clinical manifestations such as papules and pustules, and some acne patients also have papules and erythema at the same time, rosacea is often easily to be missed or misdiagnosed when acne and rosacea coexist. Although the causative association between rosacea and acne is still unclear, genetics, microbiomes, innate and adaptive immunological dysregulation, and skin barrier dysfunction have been implicated as cross-pathogenetic factors^5^. To explore the common pathogenesis of rosacea and acne, we used the datasets from the Gene Expression Omnibus (GEO) database to perform related analyses to identify the common DEGs, pathways and immune mechanisms of these two diseases. Additionally, we screened out the hub genes and transcription factors (TFs) that promote the pathogenesis of these two diseases in order to provide a novel insight into the common pathogenesis of rosacea and acne.

## Results

### Datasets processing and identification of DEGs

To maximize sample size, we pooled GSE108110^6^ and GSE53795^7^. Batch effects refer to technical differences arising from the processing and measurement of samples in different batches. To avoid batch effects having an impact on subsequent analyses, we eliminated the batch effects from the merging dataset. The boxplot revealed that the distributions of the samples in each dataset differed significantly before batch effects were eliminated, suggesting the presence of batch effects (Supplementary Fig. 1A). After eliminating the batch effect, the sample distribution of two datasets converged, with the median falling on a straight line (Supplementary Fig. 1B). Uniform Manifold Approximation and Projection (UMAP) indicated that the samples of two datasets were clustered and intertwined after the batch effect was eliminated, suggesting that the batch effect was eliminated effectively (Supplementary Fig. 1C,D). The merging dataset and GSE65914^8^ were deduplicated and averaged according to the gene name.

**Figure 1:**
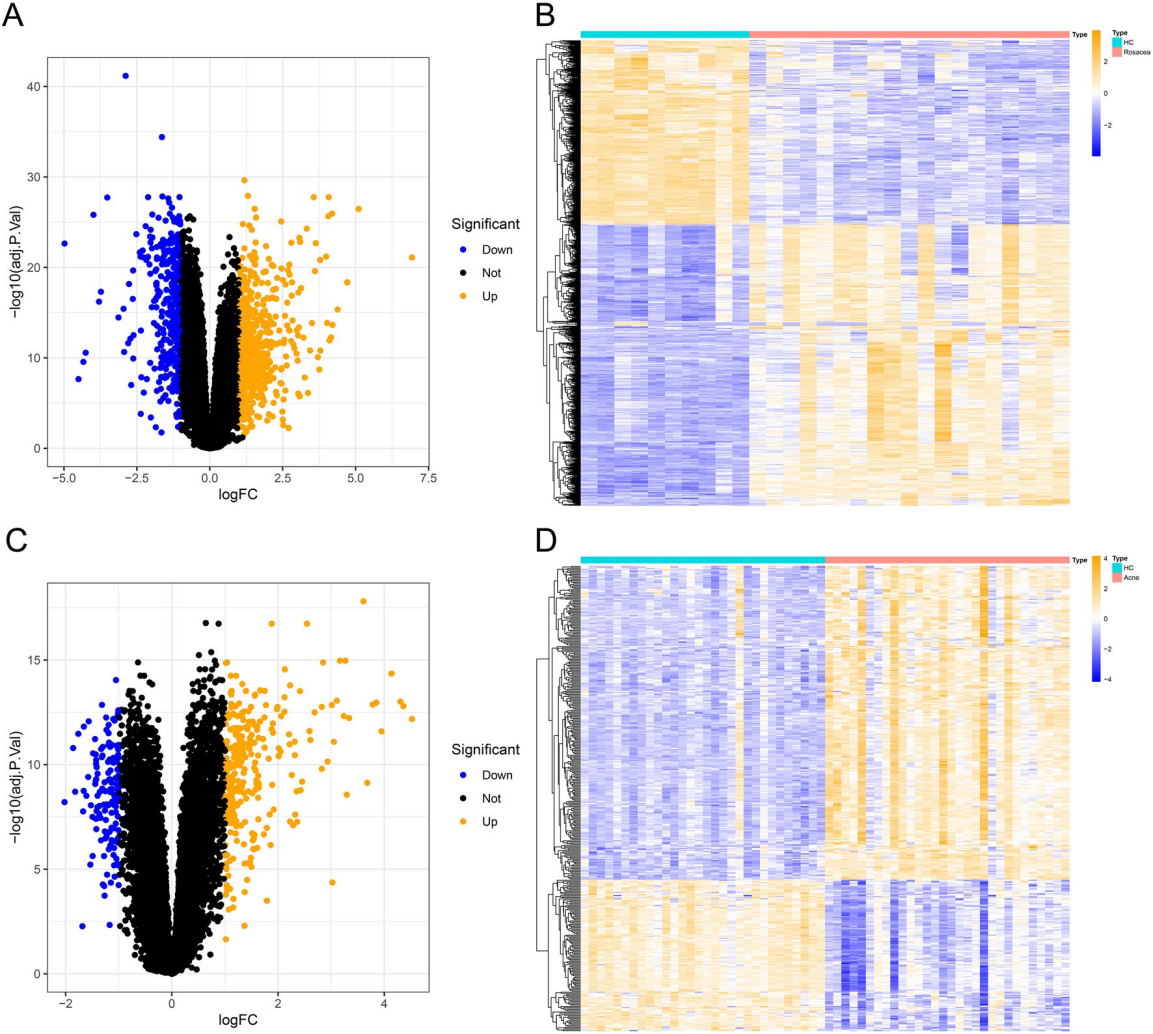
Identification of DEGs in rosacea and acne datasets. (**A**) Volcano plot of DEGs from all samples in the rosacea dataset, orange indicated higher gene expression, blue indicated lower gene expression, and black indicated genes with no significant difference in expression. (**B**) Heatmap of the DEGs in the rosacea dataset, the blue part on the left is the HC group, and the pink part on the right is the lesional group. Orange indicated higher gene expression and blue indicated lower gene expression. (**C**) Volcano plot of DEGs from all samples in the acne dataset. (**D**) Heatmap of DEGs in the acne dataset. *DEGs* differentially expressed genes, *HC* healthy control.

To identify genes that were significantly up-regulated and down-regulated in the rosacea and acne datasets, we performed differential gene expression analysis of the two datasets. We compared the expression levels of all genes in the rosacea and acne datasets and identified 913 DEGs (552 up-regulated genes and 361 down-regulated genes) and 424 DEGs (286 up-regulated genes and 138 down-regulated genes) in rosacea and acne datasets, respectively (adjusted P-value < 0.05 and |log2FC (fold change)|> 1). The volcano plot showed DEGs with adjusted P-value < 0.05 and |log2FC|> 1 (Fig. 1A,C), where orange indicated higher gene expression, blue indicated lower gene expression, and black indicated genes with no significant difference in expression. All DEGs of rosacea and acne datasets were shown as heatmaps (Fig. 1B,D). Each column in the heatmaps represented a sample, the blue part on the left is the healthy control (HC) group, and the pink part on the right is the lesional group. Each row represented a gene, where orange indicated higher gene expression and blue indicated lower gene expression, displaying rough separation of lesional and HC groups.

### WGCNA of GSE65914

**Figure 2:**
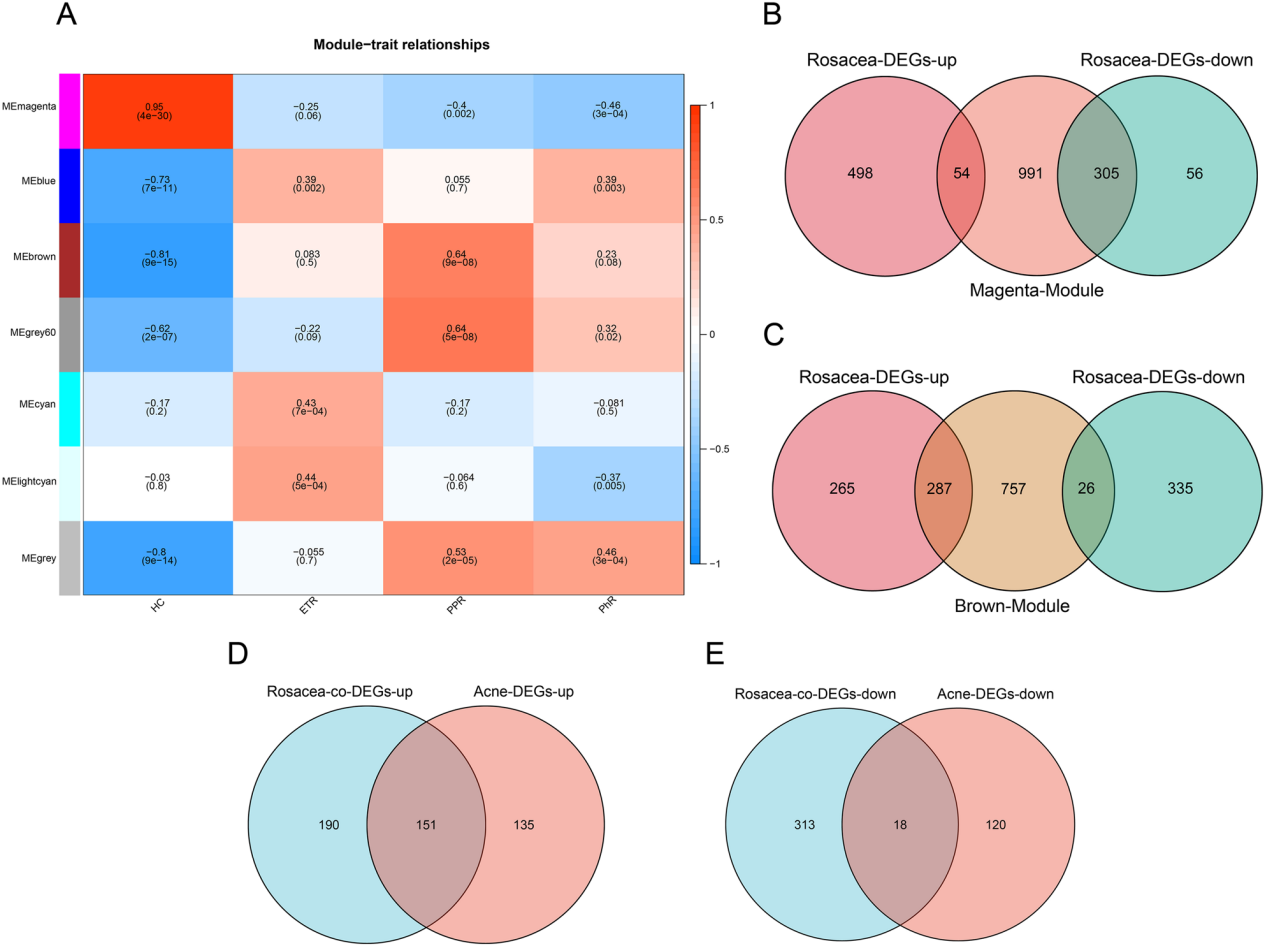
Weighted gene co-expression network analysis for GSE65914. (**A**) Heatmap of the association between rosacea and gene co-expression modules. Numbers at the top are the correlation coefficients, numbers at the bottom brackets are the corresponding P-values. (**B**) Venn diagram of the DEGs of rosacea and genes in the magenta module. (**C**) Venn diagram of the DEGs of rosacea with genes in the brown module. (**D**) Venn diagram of the up-regulated rosacea-co-DEGs and the up-regulated DEGs of acne. (**E**) Venn diagram of the down-regulated rosacea-co-DEGs and the down-regulated DEGs of acne. *DEGs* differentially expressed genes, *HC* healthy control, *ETR* erythematotelangiectatic rosacea, *PPR* papulopustular rosacea, *PhR* phymatous rosacea.

To further identify the hub genes of the rosacea dataset, weighted gene co-expression network analysis (WGCNA) was performed on GSE65914. In the results of our study, soft thresholding power (β) was selected as 6. Figure 2A showed the correlation between rosacea and gene co-expression modules. We found that the magenta and brown modules had the strongest correlation with rosacea. There were 1350 genes in the magenta module and 1070 genes in the brown module. Then, we took the intersection of the DEGs of rosacea dataset with genes in the magenta module and the DEGs of rosacea dataset with genes in the brown module separately, a total of 672 rosacea-co-DEGs were identified (Fig. 2B,C). Subsequently, we took the intersection of rosacea-co-DEGs and the DEGs of acne dataset, a total of 169 co-DEGs were identified, including 151 up-regulated genes and 18 down-regulated genes (adjusted P-value < 0.05) (Fig. 2D,E).

### GO and KEGG pathway enrichment analysis

**Figure 3:**
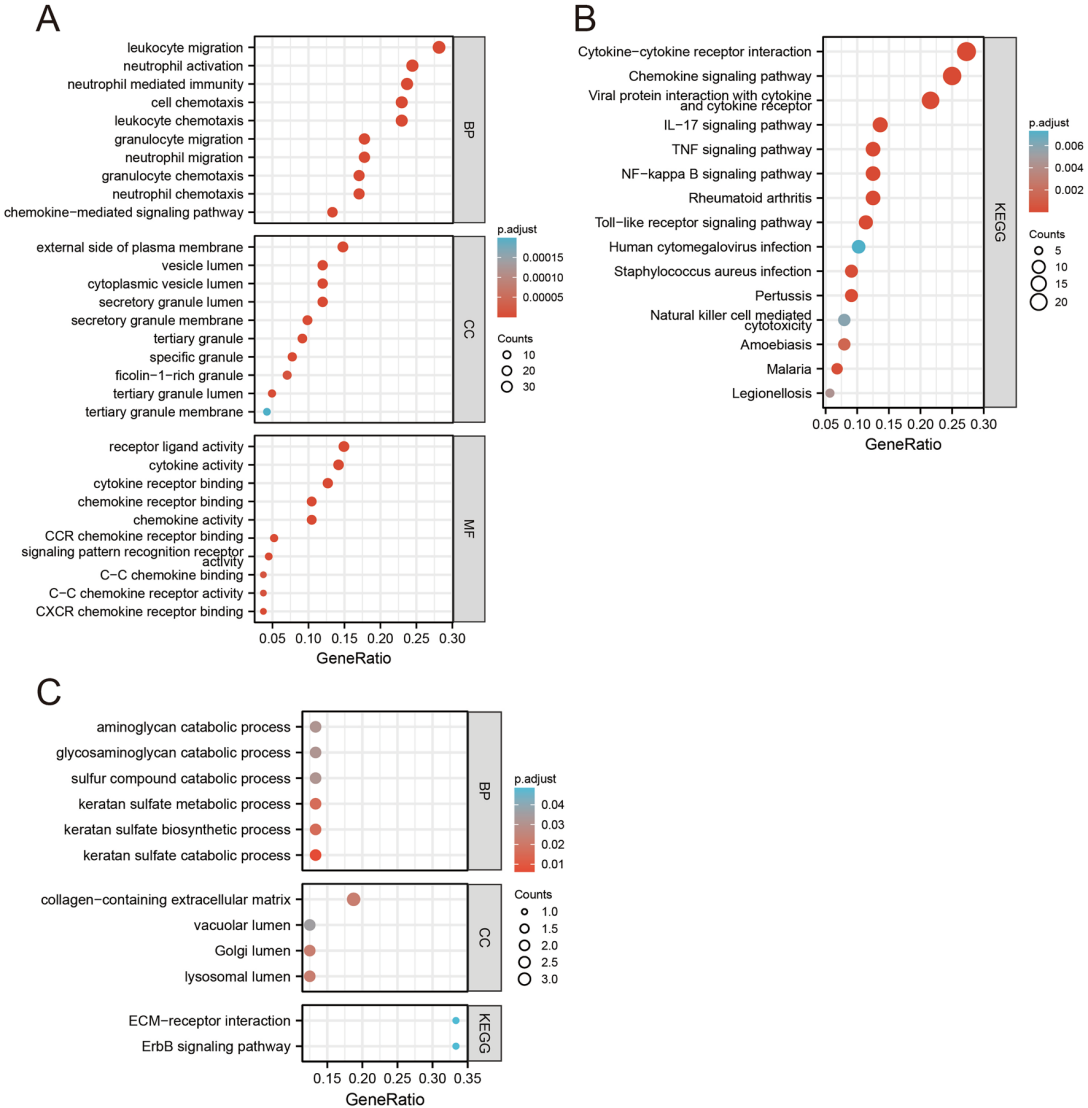
Enrichment analysis of co-DEGs. (**A**,**B**) GO and KEGG pathway enrichment analysis of the up-regulated co-DEGs. (**C**) GO and KEGG pathway enrichment analysis of the down-regulated co-DEGs. *DEGs* differentially expressed genes, *GO* gene ontology, *KEGG* Kyoto Encyclopedia of Genes and Genomes.

To identify the functions and the pathways enriched by the genes which we interested in, Gene Ontology (GO) and Kyoto Encyclopedia of Genes and Genomes (KEGG) pathway enrichment analysis were performed for rosacea-co-DEGs (Supplementary Fig. 2), DEGs of acne dataset (Supplementary Fig. 3), and co-DEGs (Fig. 3), respectively. Here, we focused on illustrating the results of the enrichment analysis of co-DEGs. The GO pathways of the up-regulated co-DEGs were mainly enriched in leukocyte chemotaxis, neutrophil migration, leukocyte migration, etc. (Fig. 3A). While the GO pathways of down-regulated co-DEGs were mainly enriched in keratan sulfate catabolic process, keratan sulfate biosynthetic process, keratan sulfate metabolic process, etc. (Fig. 3C). Furthermore, KEGG pathway analysis demonstrated that up-regulated co-DEGs participated in chemokine signaling pathway, cytokine-cytokine receptor interaction, IL-17 signaling pathway, rheumatoid arthritis, NF-κB signaling pathway, TNF signaling pathway, and Toll-like receptor signaling pathway (Fig. 3B). While down-regulated co-DEGs participated in ErbB signaling pathway and ECM-receptor interaction (Fig. 3C).

### Construction of PPI network and identification of hub genes

**Figure 4:**
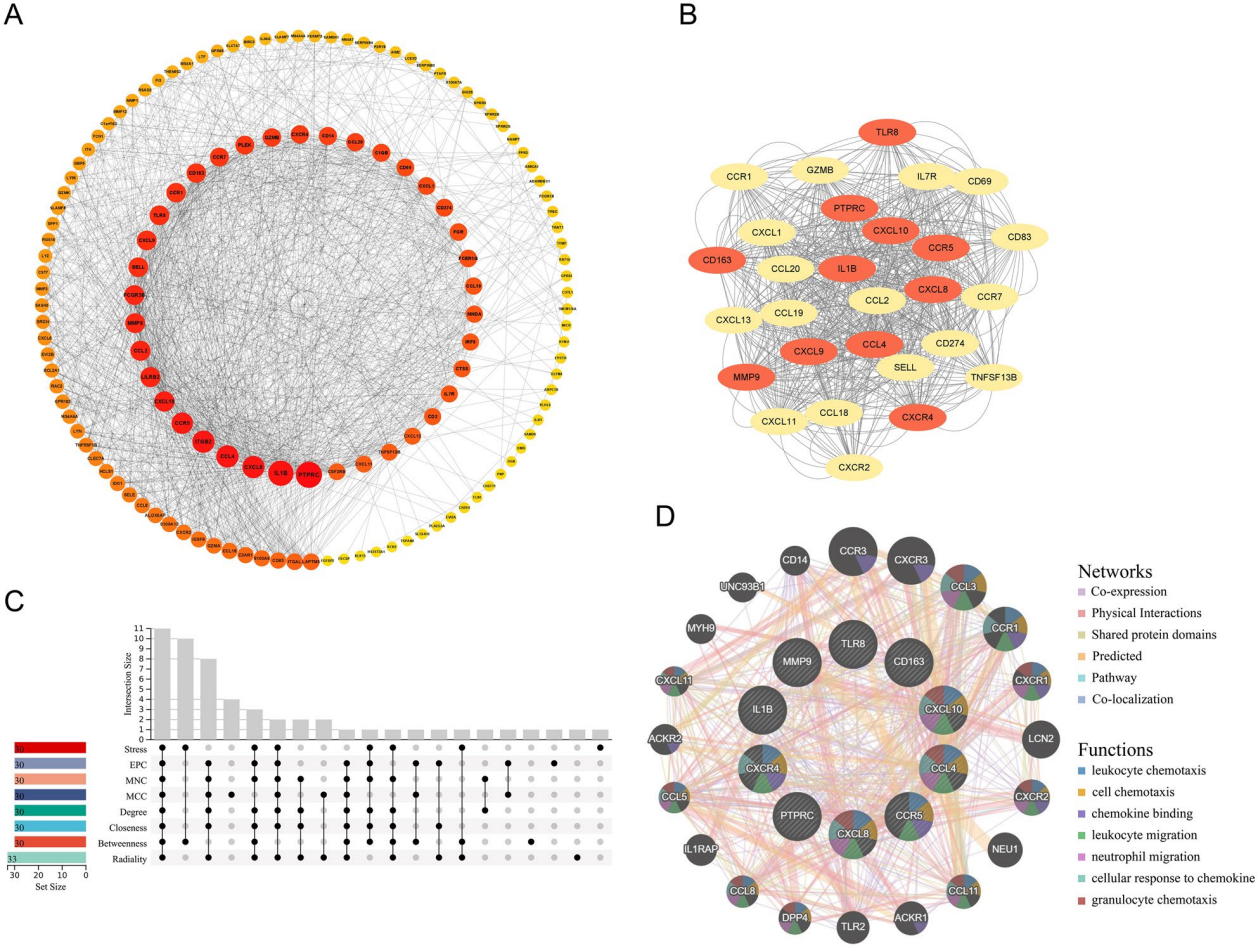
PPI network of co-DEGs, screening and constructing co-expression network of hub genes. (**A**) PPI network of co-DEGs. (**B**) The cluster with the highest score selected by the MCODE plug-in, hub genes were marked in red. (**C**) Hub genes were screened out using 8 algorithms. (**D**) Co-expression network of hub genes. PPI, protein–protein interaction.

In order to further identify the common hub genes of rosacea and acne, the protein–protein interaction (PPI) network of co-DEGs was constructed and visualized (Fig. 4A). The genes in the intersection of eight commonly used algorithms in cytoHubba plug-in were identified as hub genes (Fig. 4C). Then, based on the PPI network, we identified 11 hub genes, including IL1B, PTPRC, CXCL8, MMP9, CCL4, CXCL10, CD163, CCR5, CXCR4, TLR8, and CXCL9. Their full names and functions were listed in Supplementary Table 1. Figure 4B was the cluster with the highest score selected by the MCODE plug-in, all 11 hub genes were marked in red.

### Verification of hub genes in GSE6475 and correlation between hub genes

**Figure 5:**
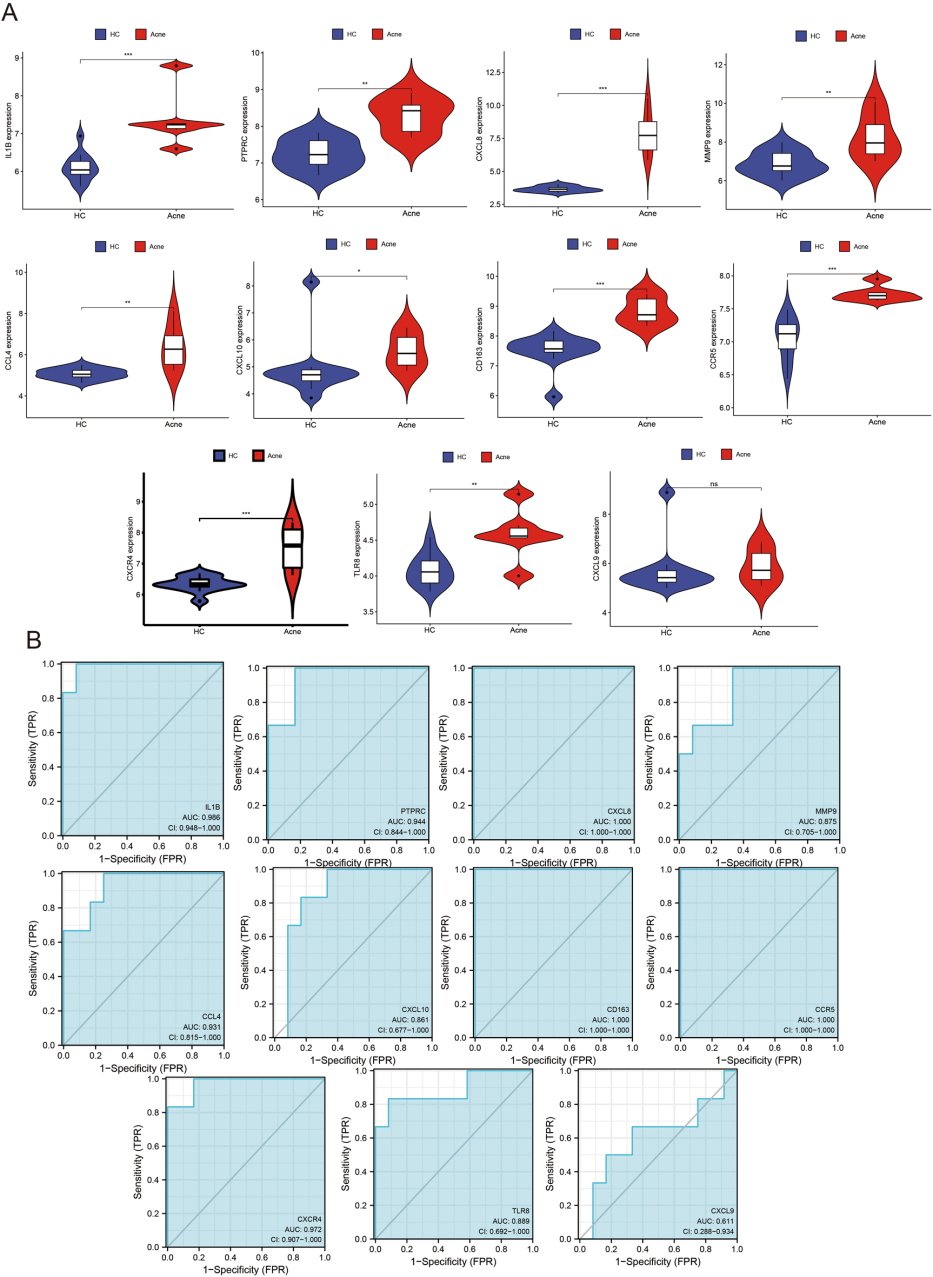
Verification of hub genes in GSE6475. (**A**) Expression levels of 11 hub genes in the lesional and HC groups in GSE6475. (**B**) The diagnostic effectiveness of 11 hub genes. P-value < 0.05 was considered statistically significant. ***p < 0.001, **p < 0.01, *p < 0.05, *ns* p ≥ 0.05. *HC* healthy control.

**Figure 6:**
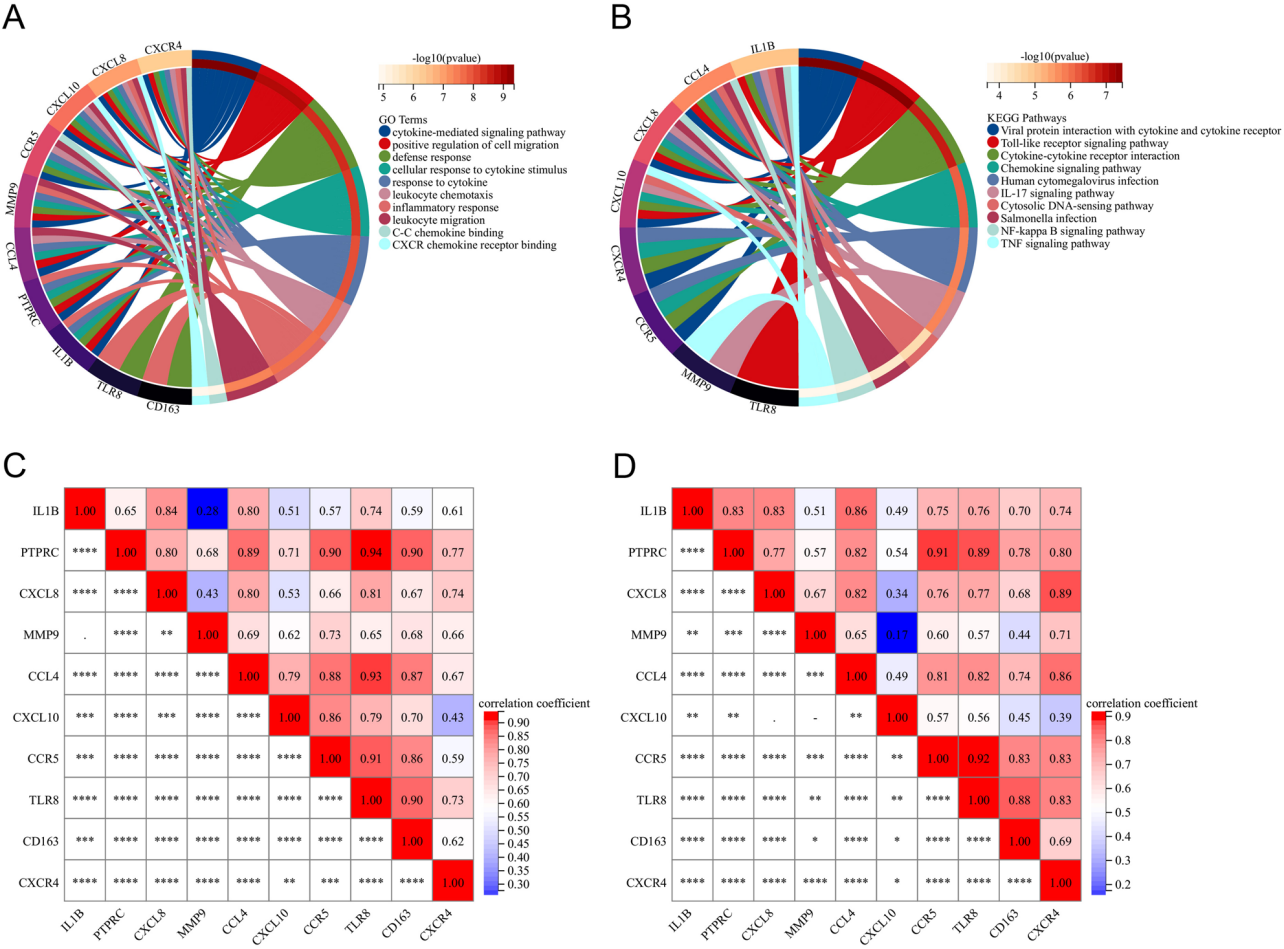
Enrichment analysis of hub genes and correlation between hub genes. (**A**,**B**) GO and KEGG pathway enrichment analysis of hub genes. (**C**) Correlation between hub genes in lesional samples of rosacea. (**D**) Correlation between hub genes in lesional samples of acne. *GO* gene ontology, *KEGG* Kyoto Encyclopedia of Genes and Genomes.

To verify the hub genes, the expression levels and ROC analysis of 11 hub genes were performed in GSE6475^9^ (Fig. 5). Genes with significantly different expression levels (P-value < 0.05) and an AUC > 0.8 were regarded as hub genes. Finally, IL1B, PTPRC, CXCL8, MMP9, CCL4, CXCL10, CD163, CCR5, CXCR4, and TLR8 were identified, and the corheatmap showed that there was a positive strong correlation between all hub genes in rosacea and acne lesions (Fig. 6C,D).

The co-expression network of hub genes was shown in Fig. 4D. GO pathway enrichment analysis revealed that these hub genes were mainly enriched in cytokine-mediated signaling pathway, cellular response to cytokine stimulus, response to cytokine, etc. (P-value < 0.05) (Fig. 6A). KEGG pathway enrichment analysis revealed that they were mainly involved in viral protein interaction with cytokine and cytokine receptor, Toll-like receptor signaling pathway, etc. (P-value < 0.05) (Fig. 6B).

### Correlation between hub genes and pathways

**Figure 7:**
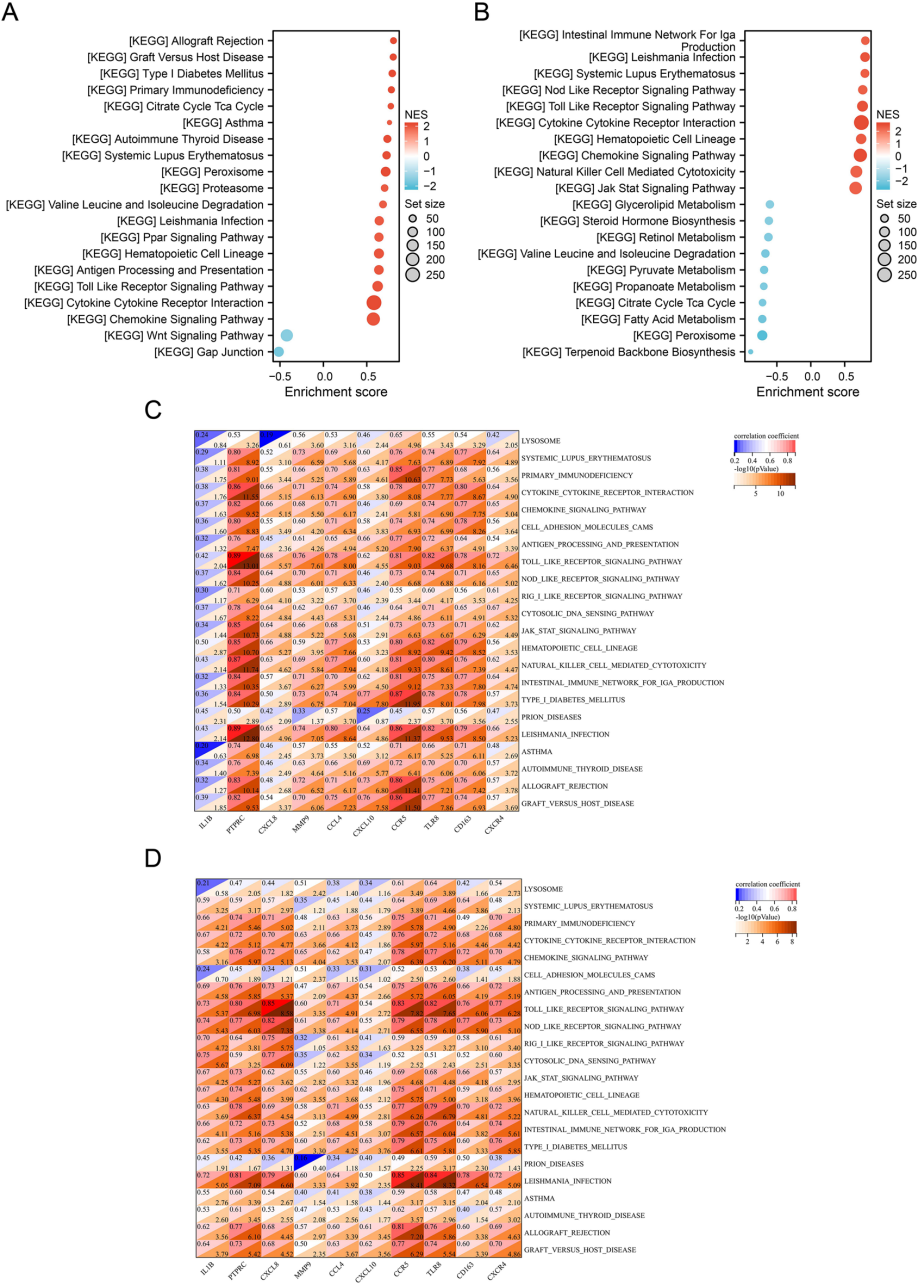
Correlation between hub genes and pathways. (**A**) The result of the GSEA of the KEGG pathway in rosacea dataset. (**B**) The result of the GSEA of the KEGG pathway in acne dataset. (**C**) Correlation heatmap between the expression of hub genes and the GSVA scores of the common pathways in rosacea dataset. (**D**) Correlation heatmap between the expression of hub genes and the GSVA scores of the common pathways in acne dataset. *GSEA* gene set enrichment analysis, *KEGG* Kyoto Encyclopedia of Genes and Genomes; *GSVA* gene set variation analysis.

To explore the relationship between the hub genes and pathways, the gene set enrichment analysis (GSEA) of the KEGG pathway was performed in the rosacea and acne datasets separately based on the expression of all genes in the two datasets, and the common GSEA pathways were identified. The GSEA of the KEGG pathway revealed that there were 37 activated and 2 suppressed pathways in the rosacea dataset, and there were 42 activated and 17 suppressed pathways in the acne dataset (P-value < 0.05, q-value < 0.25). Only the activated and suppressed pathways with the highest normalized enrichment scores were shown in the Fig. 7A,B. There were 22 common GSEA pathways in the rosacea and acne datasets, all of which were activated pathways. Supplementary Figure 5 showed that there was a clear separation of the gene set variation analysis (GSVA) scores for common GSEA pathways between the lesional and HC groups. Based on the expression of hub genes and the GSVA scores of common GSEA pathways in each sample, the relationship between hub genes and pathways were calculated. Figure 7C,D showed that all hub genes were significantly positively correlated with cytokine-cytokine receptor interaction, chemokine signaling pathway, Toll-like receptor signaling pathway, NOD-like receptor signaling pathway, JAK/STAT signaling pathway, etc. (P-value < 0.05).

### Immune cell infiltration analysis by CIBERSORT

**Figure 8:**
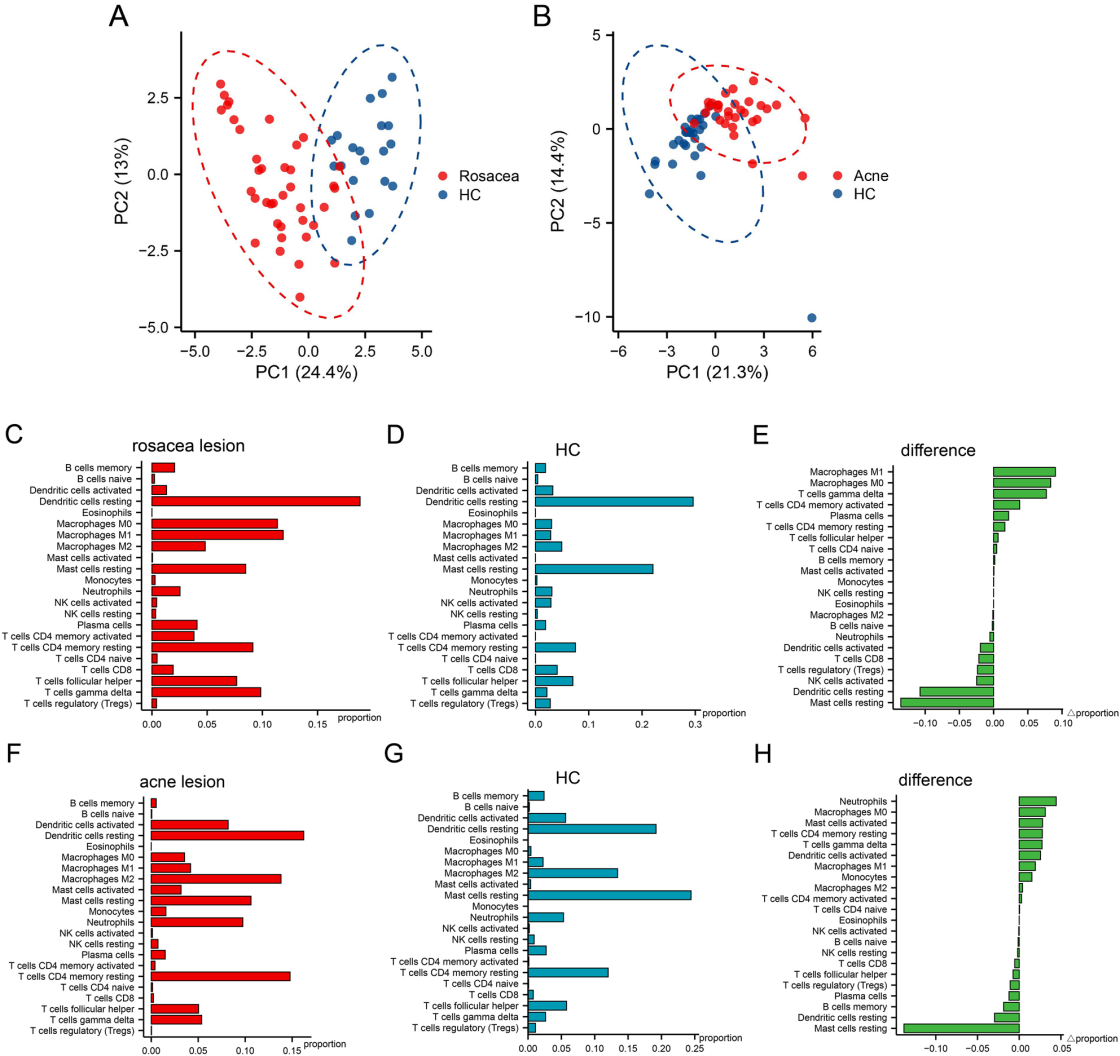
PCA cluster plot and the infiltration of immune cells among lesional and HC groups. (**A**) PCA cluster plot of immune cell infiltration in rosacea lesional and HC groups. (**B**) PCA cluster plot of immune cell infiltration in acne lesional and HC groups. (**C**,**D**) The proportions of 22 immune cells in rosacea lesional and HC groups separately. (**E**) The difference of the proportions of 22 immune cells between rosacea lesional and HC groups. (**F**,**G**) The proportions of 22 immune cells in acne lesional and HC groups separately. (**H**) The difference of the proportions of 22 immune cells between acne lesional and HC groups. *PCA* principal component analysis, *HC* healthy control.

To explore the infiltration of 22 immune cells in each sample and identify the immune cells associated with the pathogenesis of rosacea and acne, the immune cell infiltration analysis were performed on all samples of rosacea and acne datasets. Principal component analysis (PCA) cluster plot depicted that there were significant differences in immune cell infiltration between lesional and HC groups of rosacea and acne datasets (Fig. 8A,B). The heatmap showed that the distribution of 22 immune cells in all samples of rosacea and acne datasets (Supplementary Fig. 4A,B). The barplot showed the relative percentage of 22 immune cells in all samples (Supplementary Fig. 4C,D). The average proportions of each type of immune cell and the difference between lesional and HC groups were calculated (Fig. 8C–H). Compared with HC, the proportions of infiltrated M1 macrophages, M0 macrophages, gamma delta T cells, and activated memory CD4 + T cells increased most in rosacea lesions, while resting mast cells and resting dendritic cells decreased most. Meanwhile, compared with HC, the proportions of infiltrated neutrophils, M0 macrophages and activated mast cells increased most in acne lesions, while resting mast cells and resting dendritic cells decreased most.

**Figure 9:**
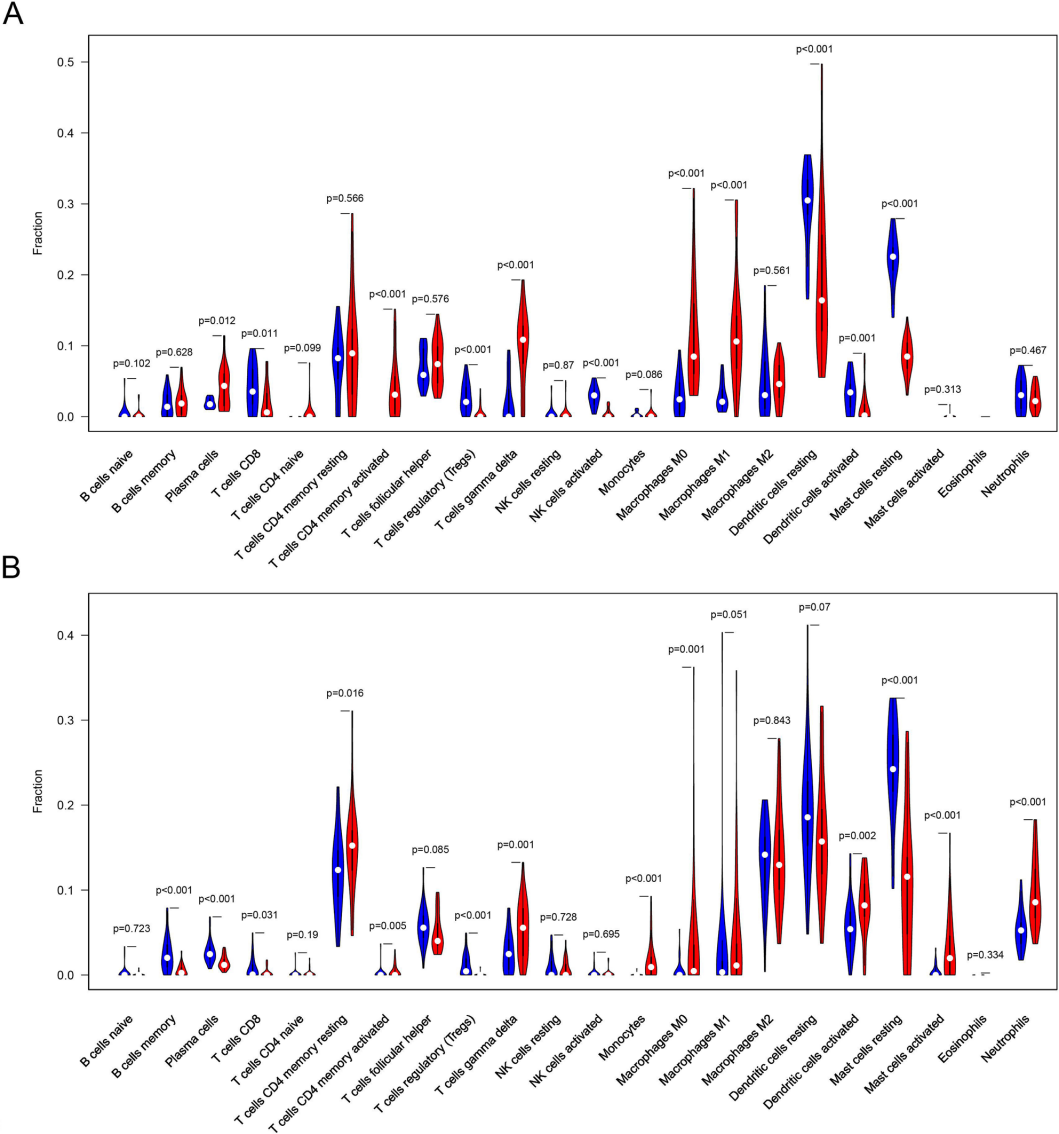
The difference of immune cells infiltration between lesional and HC groups. (**A**) The difference of immune cells infiltration between rosacea lesional (red) and HC groups (blue). (**B**) The difference of immune cells infiltration between acne lesional (red) and HC groups (blue). *HC* healthy control.

The violin plot revealed that activated memory CD4 + T cells, M0 macrophages, M1 macrophages, and gamma delta T cells infiltrated significantly more in rosacea lesional group than HC group, while Tregs, activated NK cells, resting dendritic cells, and resting mast cells infiltrated significantly less (P-value < 0.001) (Fig. 9A). Neutrophils, monocytes, and activated mast cells infiltrated significantly more in acne lesional group than HC group, while memory B cells, plasma cells, Tregs, and resting mast cells infiltrated significantly less (P-value < 0.001) (Fig. 9B).

The Corheatmap revealed that there was a positive strong correlation between resting NK cells and activated dendritic cells, activated mast cells and Tregs, monocytes and M1 macrophages, while there was a negative correlation between gamma delta T cells and resting dendritic cells, activated memory CD4 + T cell and follicular helper T cells in rosacea lesions (Supplementary Fig. 6A). And there was a positive strong correlation between activated NK cells and Tregs, activated memory CD4 + T cells and gamma delta T cells, neutrophils and activated mast cells, whereas there was a negative correlation between activated mast cells and resting mast cells, neutrophils and resting mast cells in acne lesions (Supplementary Fig. 6B).

### Correlation of the hub genes and immune cells

**Figure 10:**
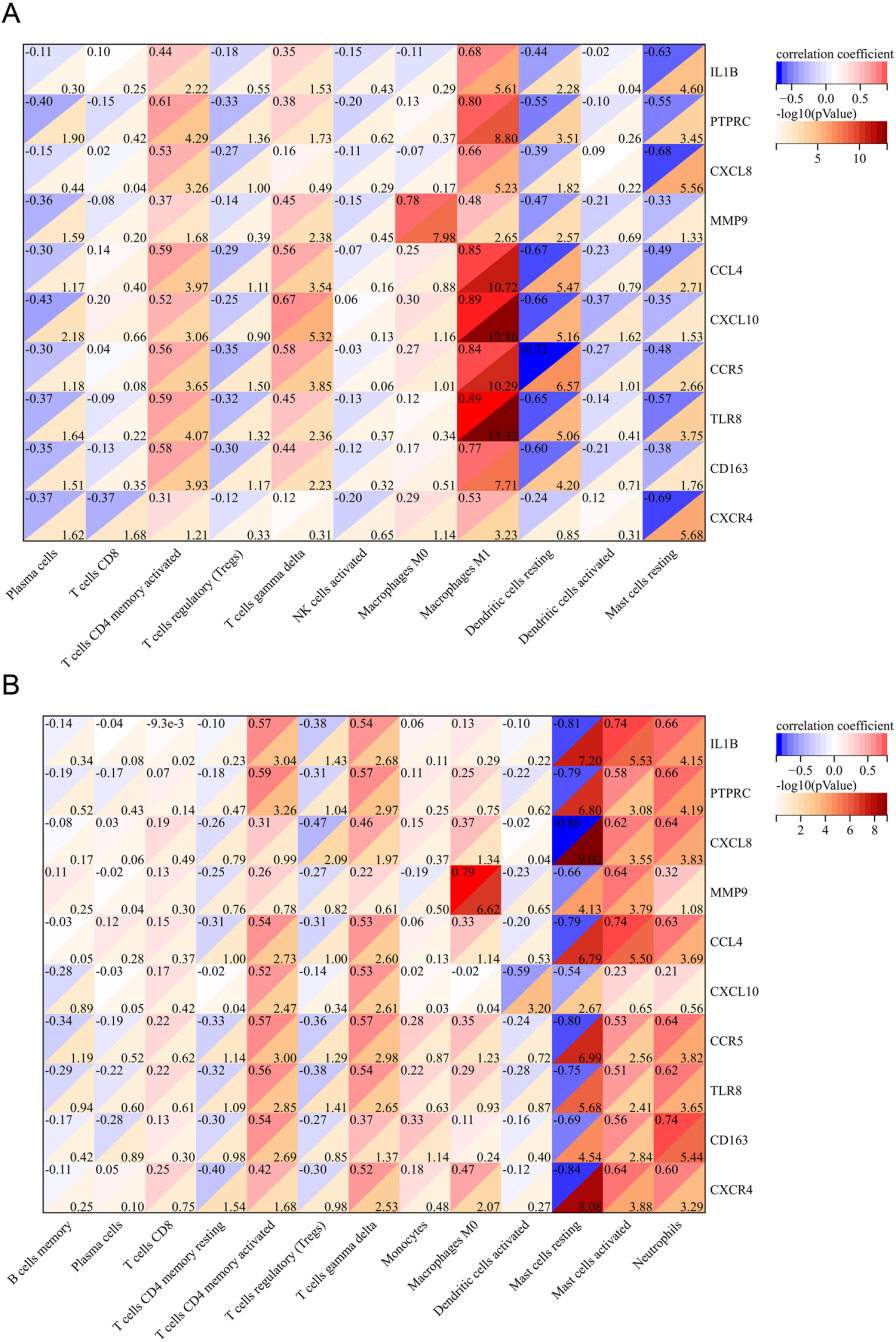
The correlation between hub genes and immune cells. (**A**) The heatmap of the correlation between hub genes and immune cells in rosacea lesions. (**B**) The heatmap of the correlation between hub genes and immune cells in acne lesions.

Subsequently, the correlation of 10 hub genes with immune cells whose infiltration was significantly different between lesional and HC groups was explored. In rosacea lesions, the results showed that all hub genes were negatively correlated with resting mast cells, and 9 (except CXCR4) were negatively correlated with resting dendritic cells. All hub genes had a positive correlation with M1 macrophages, 9 (except CXCR4) and 8 (except CXCL8 and CXCR4) hub genes had a positive correlation with activated memory CD4 + T cells and gamma delta T cells, respectively (Fig. 10A). In acne lesions, all hub genes were negatively correlated with resting mast cells, whereas 9 (except CXCL10), 9 (except MMP9) and 8 (except MMP9 and CXCL10) hub genes were positively correlated with activated mast cells, gamma delta T cells, and neutrophils, respectively (Fig. 10B). And notably, MMP9 was significantly positively correlated with M0 macrophages in both rosacea and acne lesions (R > 0.75, P-value < 0.01). The correlation between all hub genes and these immune cells was shown in the lollipop chart (Supplementary Figs. 7, 8).

### Prediction and verification of TFs

**Figure 11:**
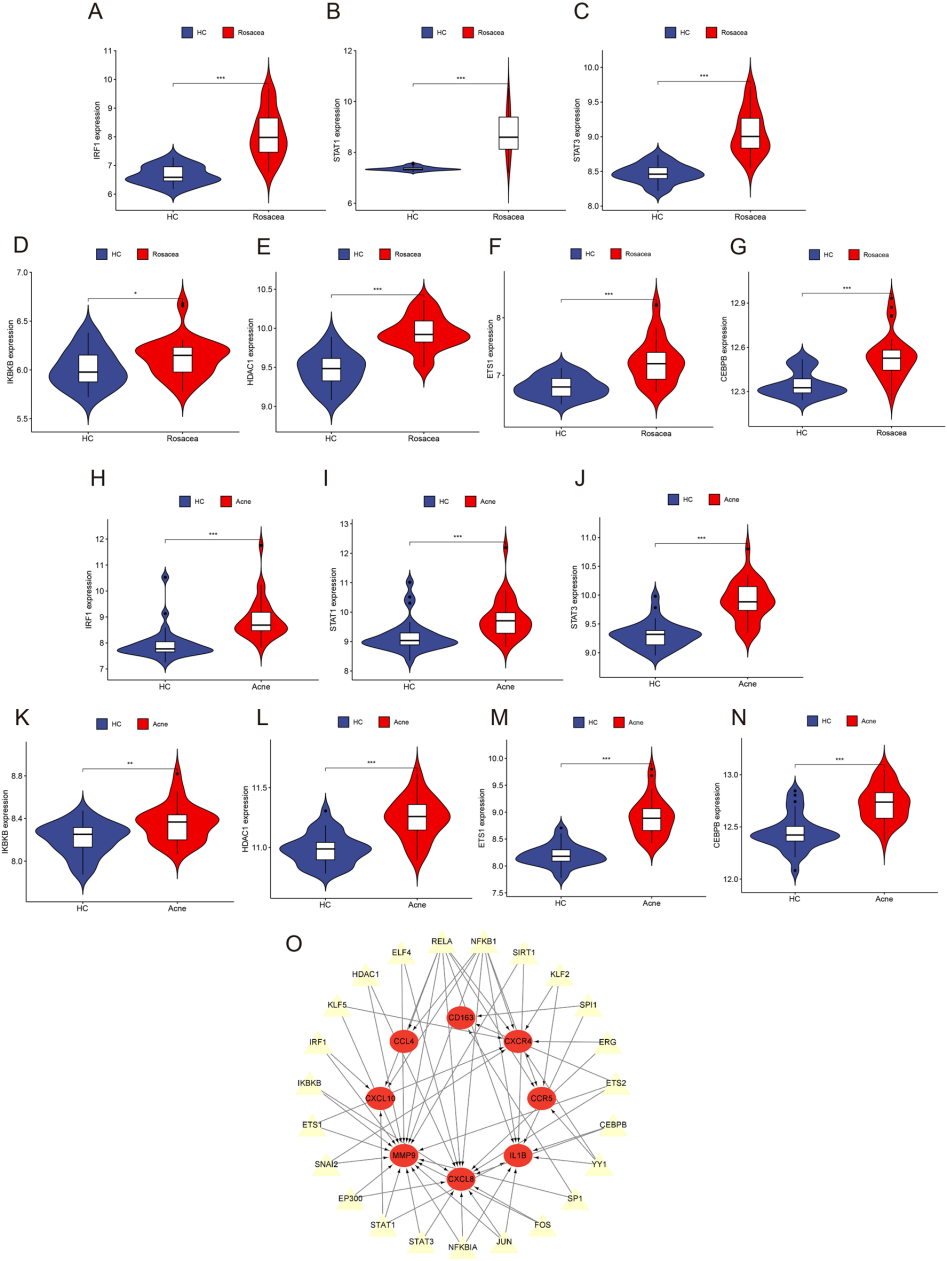
Prediction and verification of TFs, and transcriptional regulatory network. (**A**–**G**) The expression levels of IRF1, STAT1, STAT3, IKBKB, HDAC1, ETS1, and CEBPB in rosacea dataset. P-value < 0.05 was considered statistically significant. (**H**–**N**) The expression levels of IRF1, STAT1, STAT3, IKBKB, HDAC1, ETS1, and CEBPB in acne dataset. P-value < 0.05 was considered statistically significant. (**O**) Transcriptional regulatory network. Hub genes were marked in red, TFs were marked in yellow. TFs, transcription factors.

To identify the TFs that may regulate the expression of the hub genes, we identified 23 TFs based on the TRRUST database (Supplementary Table 2). A total of 9 TFs were significantly differentially expressed in rosacea and acne datasets. Compared with HC, IRF1, STAT1, STAT3, IKBKB, HDAC1, ETS1, and CEBPB were highly expressed in rosacea and acne lesions (P-value < 0.05) (Fig. 11A–N). The expression of CXCL10, MMP9, IL1B, CXCL8, and CXCR4 were co-regulated by these TFs (Fig. 11O).

## Discussion

Rosacea and acne are the most common inflammatory chronic skin diseases. Clinically, we frequently see acne patients with rosacea or patients who develop from acne to rosacea. Genetics, microbiomes, innate and adaptive immunological dysregulation, and skin barrier dysfunction have been implicated as shared pathogenetic factors in both rosacea and acne^5^. When rosacea and acne coexist, rosacea is frequently missed or misdiagnosed due to the similarity of their clinical manifestations. However, few studies have investigated their relationship. The purpose of this study was to explore the relationship between rosacea and acne. In this study, hub genes were identified by screening DEGs and constructing weighted gene co-expression and PPI networks. And TFs that regulate the expression of these hub genes have been identified. Finally, we performed immune cell infiltration analysis to compare the immune cell infiltration characteristics of rosacea and acne lesions.

A total of 151 up-regulated and 18 down-regulated co-DEGs were identified. KEGG pathway analysis of up-regulated co-DEGs demonstrated that the they were mainly involved in IL-17 signaling pathway, NF-κB signaling pathway, Toll-like receptor signaling pathway, and TNF signaling pathway. This is consistent with previous findings that these pathways are involved in the development of both rosacea and acne^7,10–15^. 10 co-DEGs, IL1B, PTPRC, CXCL8, MMP9, CCL4, CXCL10, CD163, CCR5, CXCR4, and TLR8, were identified as hub genes. The GO and the KEGG pathways analysis of hub genes revealed that they were mainly involved in immunological and inflammatory processes. And 14 pathways were significantly positively correlated with hub genes in both rosacea and acne datasets. The expression of CXCL10, MMP9, IL1B, CXCL8, and CXCR4 were co-regulated by IRF1, STAT1, STAT3, IKBKB, HDAC1, ETS1, and CEBPB, which were highly expressed in rosacea and acne lesions. Moreover, MMP9 was significantly positively correlated with M0 macrophages in both rosacea and acne lesions. The infiltration of gamma delta T cells was significantly increased and positively correlated with almost all hub genes in both rosacea and acne, suggesting that gamma delta T cells may play a crucial role in the pathogenesis of these two diseases.

Interleukin (IL)-1β is a potent proinflammatory cytokine which belongs to the IL-1 family. Previous studies have indicated that IL-1β plays a crucial role in the development of inflammation in rosacea and acne. Meng et al. found that the expression of IL-1β in lesions of rosacea patients was significantly higher than age- and sex-matched healthy volunteers, especially PPR. In acne, all patients with inflammatory acne have dermal and epidermal IL-1β expression, and the expression level correlates with disease severity^16^. Ultraviolet B and *P. acnes* are triggers for IL-1β secretion, and the inflammation induced by *P. acnes* causes the massive secretion of IL-1β from monocytes^17–19^.

Matrix metalloproteases 9 (MMP9) is a member of the MMPs family that can degrade and remodel extracellular matrix proteins^20^. MMPs mediate the rupture of the pilosebaceous follicle, whereby promotes the exacerbation of inflammation^21^. The level of MMP9 expression is significantly increased in the lesions and serum of rosacea patients^22,23^. In addition, the expression of MMP9 in the dermis of granulomatous rosacea lesions is significantly higher than that of non-granulomatous rosacea lesions, especially at the granuloma’s center^24^. MMP9 induced by ultraviolet radiation has a role in the development of granuloma by promoting tissue remodeling and enhancing recruitment of inflammatory cells into the granuloma^24^. MMP9 also participates in various cellular processes, including angiogenesis and vasodilation^20,25^. Additionally, MMP9 contributes to the progression of acne. Kang et al. found that the expression level of MMP9 in acne lesions was significantly higher than in non-lesional skin^26^. As one of the major pathogeneses of acne, *P. acne* was reported to induce the production of MMP9. Isotretinoin, quercetin, and daylight photodynamic therapy are all effective acne therapies that have been shown to decrease MMP9 expression^27–29^. MMP9 induces the production of pro-inflammatory cytokine, participates in angiogenesis, vasodilation, and tissue remodeling, which may make it the common hub gene in rosacea and acne.

CD163 is a macrophage-specific protein that is a member of group B of the scavenger receptor cysteine-rich superfamily and plays a crucial role in the regulation of the immune response^30,31^. The upregulation of CD163 is one of the major alterations in the macrophage transition to alternate activated phenotypes during inflammation^30^. In rheumatoid arthritis, psoriasis, atopic dermatitis (AD), and rosacea, CD163 levels have been shown to be elevated^32–37^. In a study including 50 rosacea patients, significant overexpression of CD163 was observed in the skin samples of PPR patients compared to the age- and sex-matched healthy volunteers^32^. In addition, CD163^+^ macrophages also play a crucial role in the progression of atherosclerosis (AS). Both rosacea and acne are closely related to AS. Several studies have shown a higher risk of AS in rosacea patients^38–40^. Massive uptake of oxidized lipid by macrophages and neutrophils (called foam cells) is believed to contribute to the common pathogenesis of acne and AS^41^. In AS, CD163^+^ macrophages are associated with plaque progression, microvascularity, and a high level of HIF1α (hypoxia-inducible factor 1α) and VEGF-A (vascular endothelial growth factor-A) expression^42^. Therefore, CD163^+^ macrophages may contribute to the development of AS in rosacea and acne patients.

Toll-like receptor 8 (TLR8), a type I transmembrane protein localized on the surface of endosomes, is expressed in monocytes, macrophages, myeloid dendritic cells, and neutrophils^43^. TLR8 induces NF-κB activation via the myeloid differentiation factor 88 (MyD88)-mediated signaling pathway and promotes the production of inflammatory factors^44^, which is crucial for innate immunity^43^. Previous studies demonstrated that TLR8 is involved in inflammatory dermatosis and autoimmune diseases, which is in the upstream of NF-κB-NLRP3 and associated with the production of IL-1β, interferon (IFN)-α, and IL-6^45–48^. However, the role of TLR8 in the pathogenesis of rosacea and acne requires further exploration.

CXCL8, CXCL10, CCL4, CCR5, and CXCR4 all belong to the chemokine superfamily and play important roles in inflammatory processes and immune responses^49^. CXCL10 is a member of the ELR^-^CXC subfamily chemokines, which play a crucial role in a variety of autoimmune diseases^50^. CXCL10 binds to its ligand CXCR3 to guide CXCR3^+^ effector CD4^+^ and CD8^+^ T cells selectively migrated to autoimmune sites and tumor sites^51^. CXCL10 and CXCR3 were found in keratinocytes and dermal infiltrates from active psoriasis plaques, and psoriasis patients had higher serum levels of CXCL10^52^. Buhl et al. demonstrated that the expression of CXCL10 was increased in the lesional skin of rosacea patients^8^. Similar results were observed in a cohort study, the CXCR3 ligands CXCL9, CXCL10, and CXCL11 were overexpressed in acne lesions^7^. All of these findings indicate that the expression levels of CXCL10 are elevated in rosacea and acne lesions, and its role in the pathogenesis of these two diseases requires further study. In addition, we also found that the expression level of CXCR4 is increased in these two diseases in our study. Helfrich et al. found an eightfold increase in CXCR4 expression in lesional skin from ETR patients compared to HC^53^. Su et al. demonstrated that in the mouse model of allergic contact dermatitis, the CXCL12/CXCR4 signaling pathway induces itching and pain feeling^54^. It requires further study to determine whether the CXCL12/CXCR4 signaling pathway has a role in some rosacea and acne patients with itching and pain.

By the prediction and verification analysis of TFs, we found that IRF1, STAT1, STAT3, IKBKB, HDAC1, ETS1, and CEBPB regulate the expression of hub genes in a synergistic manner. Several previous studies have demonstrated that these TFs play a crucial role in the pathogenesis of rosacea and acne. Signal Transducers and Activators of Transcription 1 (STAT1) is a member of the STAT family, it is activated by Janus kinases (JAK)^55^. STAT1 is critical in inflammatory diseases, such as systemic lupus erythematosus, inflammatory bowel disease, and psoriasis^56–58^. Activation of STAT1 leads to the upregulation of several pro-inflammatory chemokines in the epidermis, such as CXCL9, CXCL10, and CCL2^59^. Saez-de-Ocariz et al. found that rosacea is a striking feature in family members with a STAT1 gain-of-function mutation, indicating that STAT1 may contribute to the development of chronic inflammation^60^. Deng et al. performed RNA-seq on lesional skin from rosacea patients and found that the epidermal STAT1/IRF1 signature were observed in all rosacea subtypes^61^. STAT1-regulated gene transcription mediates many of the immune and inflammatory actions of IFN-γ^62^. Previous studies have implicated that IFN-γ is implicated in the pathogenesis of rosacea and acne. *P. acnes* can induce IFN-γ secretion by Th17 cells and CD4^+^ T cells^63,64^. In an in vitro experiment, acne-associated *P. acnes* phylotypes induced 2–3 times higher levels of IFN-γ in PBMCs than healthy phylotypes^65^. In addition, the expression level of IFN-γ was significantly increased in rosacea lesions, which was validated by immunocytochemistry to have a largely higher staining for IFN-γ in lesions^8^. Whether STAT1 has a role in the pathogenesis of rosacea and acne by regulating gene transcription to mediate the immune and inflammatory actions of IFN-γ remains to be confirmed in the future.

Signal Transducers and Activators of Transcription 3 (STAT3) is a component of the IL-6 activated acute phase response factor complex and is also a member of the STAT family^66^. It has been demonstrated that the IL-6/STAT3 signaling pathway plays a role in inflammation^67–69^. Wang et al. found that STAT3 and related pathways are up-regulated in rosacea, and they speculated that STAT3 expression is up-regulated in keratinocyte after skin barrier dysfunction, leading to the secretion of inflammatory factors and immune infiltration^70^. In a model in which HaCaT cells treated with the antibacterial peptide LL-37 to simulate rosacea caused by *Demodex folliculorum* (*D. folliculorum*) infection, JAK 2 and STAT3 expression levels were increased, indicating that rosacea caused by *D. folliculorum* infection may be associated with the activation of the JAK/STAT signaling pathway^71^. These evidences indicate that STAT3 may be an important gene in the pathogenesis of rosacea and acne, and it remains to be confirmed whether medications that target STAT3 may effectively treat rosacea and acne.

NF-κB is normally sequestered in the cytoplasm, bound to inhibitory κB (IκB) proteins as an inactive complex^72^. In response to lipopolysaccharide (LPS), an endotoxin recognized by the TLR4 receptor on immune cells, cellular IκB kinase (IKK) complex is activated and the cytoplasmic IκB protein is phosphorylates^73^. The phosphorylated forms of IκB are subjected to ubiquitination and then degraded in the proteasome, which frees NF-κB to translocate to the nucleus where it regulates gene transcription^74^. Then NF-κB moves into the nucleus and binds to the κB motif of inflammation and immune genes, such as IL-1β^75^. As a component of the IKK complex, IKKβ is intimately associated with the expression of NF-κB. And in our study, we found that the expression levels of IKKβ is increased in both rosacea and acne lesions, and that it may regulate the expression of hub genes. It has been demonstrated that the NF-κB signaling pathway plays a crucial role in the pathogenesis of rosacea and acne^26,76–79^. Most inflammatory stimuli, including LPS, require the IKKβ subunit for NF-κB activation^80^. Benzothiazolone derivatives have been used in the treatment of acne^81^. Kim et al. found that LYR-71, a benzothiazolone derivative, is a potent IKKβ inhibitor that prevents NF-κB activation in macrophages and may help in inhibiting the expression of inflammatory cytokines^75^. These findings suggest that IKKβ may play a role in the pathogenesis of rosacea and acne by influencing the NF-κB signaling pathway and regulating the secretion of pro-inflammatory cytokines.

In addition, we also performed immune infiltration analysis for the rosacea and acne datasets, respectively. Notably, MMP9 was significantly positively correlated with M0 macrophages in both rosacea and acne lesions in our study. And the infiltration of gamma delta T cells in rosacea and acne lesions was higher than in HC and positively correlated with the most of hub genes. However, few previous studies have reported the role of gamma delta T cells in this process.

Gamma delta T cells (also called γδT cells) are an unconventional population of T lymphocytes. They are significantly enriched in mucosal and epithelial sites, such as the skin and respiratory, digestive, and reproductive tracts^82^. γδT cells has a variety of functions, including cytokine and chemokine production, antigen-presenting functions, and regulation abilities^83^, and therefore play a crucial role in autoimmune, infection, allergy, cancer, among others^84–87^. They produce a variety of cytokines, such as IL-17 and IFN-γ^82^. IL-17-producing γδT cells induces the recruitment of neutrophils and monocytes and increases the inflammation response^82^. In a variety of immune skin diseases, including AD, alopecia areata, and psoriasis, the number of intracutaneous γδT cells has been demonstrated to be increased^88–90^. Cai et al. found that epidermal hyperplasia and inflammatory response induced by IL-23 and IMQ were significantly decreased in T cell receptor δ deficient mice^91^. These evidences suggest that γδT cells are crucial in inflammatory diseases and may be therapeutic targets in the future. As for the role of γδT cells in the progression of rosacea and acne, additional research is required.

In addition, we also found that there was a significant positive correlation between MMP9 and M0 macrophages in rosacea and acne lesions. It has been demonstrated that MMP9 expression and M0 macrophage infiltration were significantly increased in both rosacea and acne lesions^22,23,26,92^, which is consistent with our results. MMP9 can degrade extracellular matrix and plays a crucial role in the development and spread of inflammatory response^21^. M0 macrophages (resting macrophages) can be polarized into M1 macrophages (classically activated macrophages) under the stimulation of IFN-γ and LPS and into M2 macrophages (alternatively activated macrophages) under the stimulation of IL-4 and IL-13. M1 macrophages then secrete a large number of pro-inflammatory factors, which mainly promote the development of inflammation. Anti-inflammatory factors are secreted by M1 macrophages, which serve an anti-inflammatory function^93^. Inflammation in rosacea and acne can be aggravated by polarizing macrophages towards the M1 phenotype^94–97^, indicating that macrophages play a crucial role in the pathogenesis of these two diseases. Previous studies have shown a significant positive correlation between MMP9 expression and M0 macrophages infiltration in inflammation-related diseases, such as coronary artery disease (CAD), AS, adhesive capsulitis, and cancers^98–101^. However, the causal relationship between them has not yet been clarified. Linton et al. found that M0 macrophages secrete MMP9 in the early phases of pancreatic cancer to promote tumor progression^102^. Therefore, we hypothesized that M0 macrophages may secrete MMP9 and act in concert with it to promote the development of inflammatory responses in rosacea and acne.

Despite the fact that rosacea and acne frequently occur together clinically and have cross pathogenesis, it is important to note that rosacea and acne are two separate diseases with distinct pathogenesis, and their clinical manifestations are not identical. The main difference between the clinical manifestations of rosacea and acne is that rosacea patients have persistent facial erythema, flushing, a burning sensation, telangiectasia, and in some cases hyperplasia of the facial tissue^1^. Rosacea is primarily caused by the dysregulation and upregulation of the innate immune system, leading to in excessive inflammation and vasodilation. In addition, hyperreactive neurovascular and exogenous factors play a significant role in the pathogenesis of rosacea^13^. Transient receptor potential (TRP) cation channels are widely expressed on keratinocytes and endothelial cells. Activation of the TRP family of channels leads to the release of mediators of neurogenic inflammation and pain, such as substance P and calcitonin gene-related peptide. Persistent facial flushing in rosacea patients is mainly induced by these vasoregulatory neuropeptides^10^. The decreased thermal pain threshold and increased burning sensation in the skin of rosacea patients may be related to the increased activity of the TRPs. In addition, connective tissue hyperplasia in some rosacea patients is related to the persistence of inflammation, the activation of mast cells and the release of MMP1, MMP9, IL-6, and histamine, and the increased activity of the TRPs^14^. However, acne is mainly caused by follicular hyperkeratinization, excess sebum, inflammation, and *P. acnes*, which does not typically involve neurovascular dysergulation^77^. Consequently, persistent erythema, flushing, burning sensation, and telangiectasia are not typically observed in acne patients. Therefore, despite the fact that rosacea and acne share some clinical manifestations and pathogenesis, great care must be taken not to confuse these two diseases in order to avoid missed and misdiagnosis.

However, our study also has some limitations. Firstly, there is limited availability of datasets in the GEO database related to rosacea and acne. Despite pooling two acne datasets, the sample size remains small, potentially leading to instability in statistical analyses. Secondly, we were unable to identify additional rosacea datasets to verify the hub genes that have already been confirmed. Thirdly, inherent variability in biological systems may impact the analysis outcomes. Fourthly, the results of the analysis are also influenced by the quality of the raw data. Low-quality sequencing, chip data, or other experimental data may result in inaccurate analytical outcomes. Fifthly, our research conclusions need further validation in in vitro models, which will be a focus of our future studies. In addition, we believe that our study may provide ideas and directions for future research.

In summary, we identified 169 common DEGs after analyzing the datasets of rosacea and acne. Then we identified 10 hub genes that may play important roles in the pathogenesis of these two diseases by verifying in another dataset, and we also identified TFs that may regulate the expression of these hub genes. Then we found the pathogenesis of rosacea and acne had some similarity in terms of immune responses through immune cell infiltration analysis. This is the first study to explore the common hub genes and critical immune cells of rosacea and acne, which helped to further clarify the common molecular pathogenesis of these two diseases.

## Methods

### Datasets source and processing

**Figure 12:**
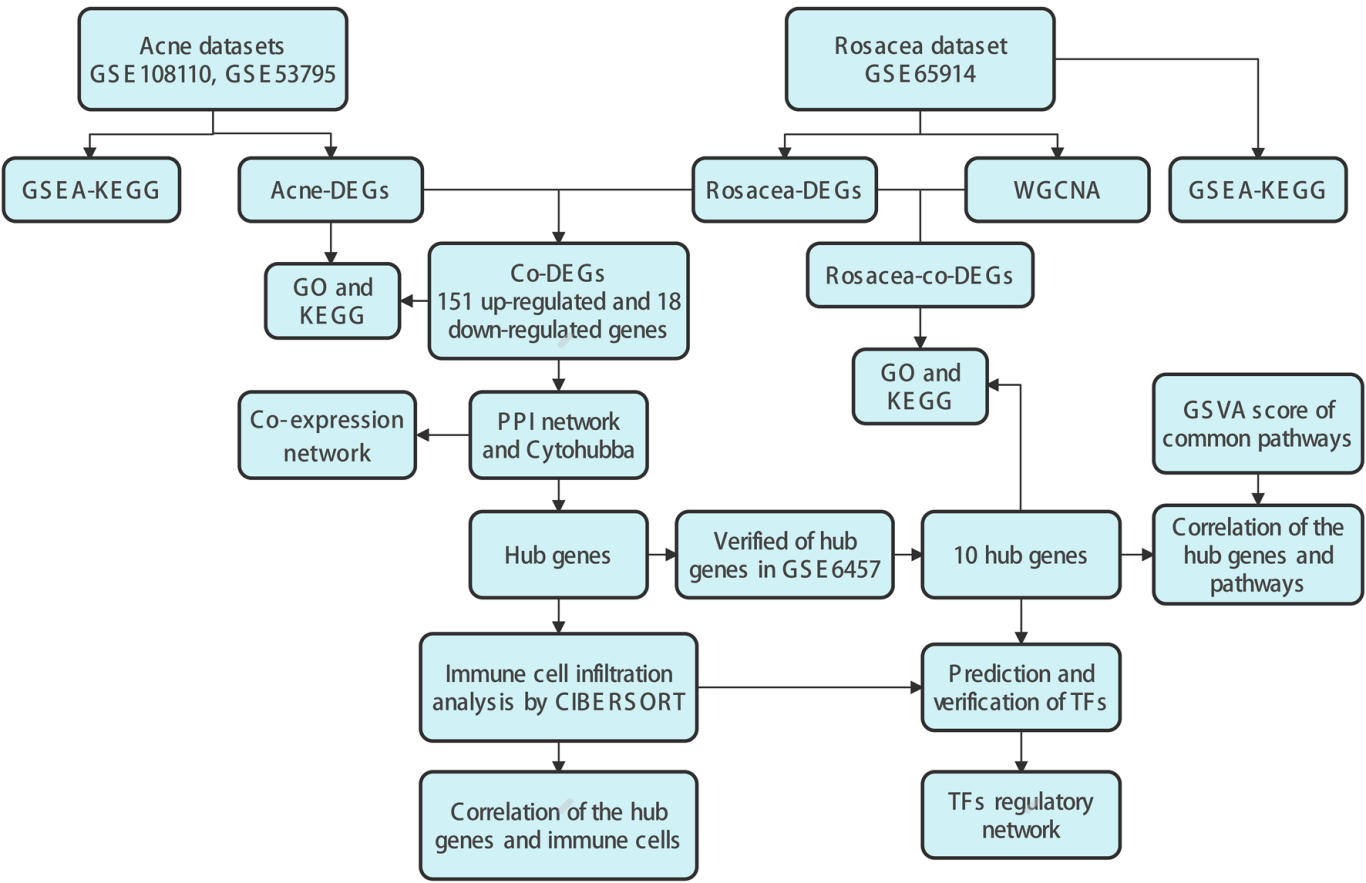
Flow chart summarizing the procedures used in this study.

The workflow is depicted in Fig. 12. Searched for “rosacea” and “acne” in the GEO database (https://www.ncbi.nlm.nih.gov/geo/) and chose datasets with the following characteristics: (a) The study includes lesional skin samples as well as non-lesional skin samples or HC skin samples. (b) The experiment type is expression profiling by array. Consequently, we identified one rosacea dataset (GSE65914) and three acne datasets (GSE108110, GSE53795, and GSE6475) meeting these criteria. To maximize sample size, we pooled GSE108110 and GSE53795, and GSE6475 was used as the verification dataset. The description of these datasets was shown in Table 1. To ensure the consistency of acne lesional samples, papule samples from GSE108110 at 21 days were excluded. We processed downloaded datasets and annotated probes with platform annotation files using R software (version: 4.2.1). Logarithmic conversion was uniformly applied to all microarrays. Each dataset’s gene expression matrix file was deduplicated and averaged according to gene name. The gene expression matrix file of GSE108110 and GSE53795 were then pooled using the “inSilicoMerging” R package. Additionally, the combat function from the “sva” R package was used to eliminate the batch effect.

**Table 1:** Description of the selected datasets.

GSE number	Platform	Experiment type	Source type	Samples			Country	Attribute	Author
				Patients	Controls	Total			
GSE65914	GPL570	Array	Human Skin	38	20	58	France	Test	Buhl et al. (2015)
GSE108110	GPL570	Array	Human Skin	18	18	36	France	Test	Carlavan et al. (2018)
GSE53795	GPL570	Array	Human Skin	12	12	24	France	Test	Kelhälä et al. (2014)
GSE6475	GPL571	Array	Human Skin	6	12	18	USA	Verification	Trivedi et al. (2006)

### Identification of DEGs

The “Limma” R package was used to identify DEGs between lesional and HC groups of the rosacea and acne datasets. The “Limma” R package is frequently employed for the identification of DEGs in both microarray and RNA-seq data. The statistical analysis is conducted using linear models and Bayesian methods. Flod change value and P-value were calculated for each gene in each sample. Adjusted P-value < 0.05 and |log2FC|> 1 were used as the cut-offs to identify DEGs. Independently, up- and down-regulated genes were obtained. The “ggplot2” and “pheatmap” R packages were used to generate the volcano plot and heatmap, respectively.

### WGCNA for GSE65914

The “WGCNA” R package was used to construct the gene co-expression network of GSE65914. Firstly, the similarity matrix was defined and the outlier samples were eliminated. CutHeight was set to 80. Soft thresholding power (β) was selected, and the similarity matrix was converted into an adjacency matrix representing the connection strength relationship between genes. Then the adjacency was converted into a topological overlap matrix and the distance between genes was calculated for hierarchical clustering based on the topological overlap dissimilarity measure. Dynamic tree-cutting algorithm was used to construct cluster dendrogram and divide modules. MinModuleSizes was set to 30, deepSplit was set to 2, and mergeCutHeight was set to 0. 25. The correlation between modules and rosacea was calculated using Pearson’s correlation analysis, and the significant module were identified. Then, using the “ggplot2” R package for visualization, we took the intersection of the DEGs of rosacea dataset and genes in the two most significant modules, regarding these genes as common DEGs in rosacea dataset, i.e., “rosacea-co-DEGs”. Finally, we took the intersection of rosacea-co-DEGs and the DEGs of acne dataset, regarding them as common DEGs, i.e., “co-DEGs”.

### GO and KEGG pathway enrichment analysis

GO and KEGG pathway enrichment analysis were performed for DEGs of acne dataset, rosacea-co-DEGs, and co-DEGs separately^103–105^. The “ClusterProfiler” R package was used for GO and KEGG pathway enrichment analysis, the entire human genome was used as universe. And the “org.Hs.eg.db” and “ggplot2” R packages were used for ID conversion and visualization, respectively.

### Construction of PPI network and identification of hub genes

Using STRING online tools (https://cn.string-db.org) and Cytoscape software (version: 3.9.1), a PPI network was constructed and visualized. MCODE plug-in was used to select the cluster with highest score. Hub genes were identified from the genes at the junction of eight commonly used algorithms in cytoHubba plug-in of Cytoscape (Maximal Clique Centrality (MCC), Maximum Neighborhood Component (MNC), Edge Percolated Component (EPC), Betweenness, Closeness, Radiality, Stress, and Degree), and the “UpSet” R package was used for visualization.

### Verification of hub genes in GSE6475 and correlation of hub genes

In GSE6475, which comprises 6 lesional samples and 12 non-lesional samples of acne, the mRNA expression of 11 hub genes was validated. First, the gene expression levels of the microarray were logarithmic transformed. The T-test was used to compare two groups, and a P-value < 0.05 was considered statistically significant. Using the “pROC” and “ggplot2” R packages, ROC analysis and visualization were performed to predict the diagnostic efficiency of hub genes. Correlation between all hub genes was calculated using Pearson’s correlation analysis, and the “corrplot” R package was used for visualization. Using Genemania online tools (http://www.genemania.org/), the co-expression network of hub genes was constructed subsequently. Then, GO and KEGG pathway enrichment analyses were performed on hub genes.

### Correlation between hub genes and pathways

The “clusterProfiler” R package was used for GSEA analysis, and the “ggplot2” R package was used for visualization. Then, we performed GSVA of the common GSEA pathways in the rosacea and acne datasets separately by using the “GSVA” R package, adjusted P-value < 0.05 and q-value < 0.25 were considered significant. The “pheatmap” R package was used to construct heatmap based on the GSVA score of the common GSEA pathways in each sample. And based on the expression of the hub genes and the GSVA scores of the common GSEA pathways in each sample, the correlation between hub genes and pathways was calculated using Pearson’s correlation analysis, and the “corrplot” R package was used for visualization. P-value < 0.05 was considered significant.

### Immune cell infiltration analysis by CIBERSORT

CIBERSORT is a computational tool for analyzing the proportion of diverse immune cells in tissues according to the known reference set LM22 (leukocyte signature matrix) based on microarray and RNA-seq data. The “CIBERSORT” R package was used to explore the infiltration of 22 immune cells in every sample. The “ggplot2” R package was used to perform PCA and visualize the results of it. Then, we visualized the distribution of 22 immune cells in all samples using the “pheatmap” R package. After that, using the “ggplot2” R package, the relative percentage of 22 immune cells in all samples was reflected via a barplot when the total infiltration of immune cells was regarded as 100%. Then, the average proportion of each type of immune cell in the lesional and HC groups was calculated, as well as the difference between the two groups. The “ggplot2” R package was used to visualize the results. Using the “vioplot” R package, the difference in infiltration of 22 immune cells between the lesional and HC groups was visualized as a violin plot. Finally, the relationship between all immune cells in lesional samples was analyzed and visualized using “corrplot” R package.

### Correlation of the hub genes and immune cells

Then, the Pearson’s correlation analysis was used to calculate the correlation between all hub genes and immune cells that infiltrated significantly differently between lesional and HC groups, and the “ggplot2” R package was used to visualize the results. In order to show the correlation between all hub genes and immune cells more intuitively, the “ggpubr” R package was used to obtain the lollipop chart.

### Prediction and verification of transcription factors

The TFs of hub genes with an adjusted P-value < 0.05 were obtained from TRRUST database (https://www.grnpedia.org/trrust). The expression levels of these TFs were then compared between the lesional and HC groups using the T-test and visualized using the “limma” and “ggpubr” R packages. P-value < 0.05 was considered significant. Cytoscape (version: 3.9.1) was used for constructing transcriptional regulatory network.

### Supplementary Information

Supplementary Information.

## Data Availability

The datasets used and/or analyzed during the current study are available from the corresponding author on reasonable request.
