# Supplementary material for: Exploring the association between rosacea and acne by integrated bioinformatics analysis
Source: Sci Rep. 2024 Feb 6;14:3065. doi: 10.1038/s41598-024-53453-x (PMC10847114; doi:10.1038/s41598-024-53453-x)
Supplement: Supplementary file 1 — Supplementary Information. [file 41598_2024_53453_MOESM1_ESM.docx]

**Supplementary Table 1** Details of the hub genes.

| **No.** | **Gene symbol** | **Full name** | **Function** |
| --- | --- | --- | --- |
| 1 | IL1B | Interleukin-1 beta | Potent proinflammatory cytokine. Initially discovered as the major endogenous pyrogen, induces prostaglandin synthesis, neutrophil influx and activation, T-cell activation and cytokine production, B-cell activation and antibody production, and fibroblast proliferation and collagen production. Promotes Th17 differentiation of T-cells. |
| 2 | PTPRC | Protein Tyrosine Phosphatase Receptor Type C | PTPRC is one of the most abundant leukocyte cell surface glycoproteins and is expressed exclusively upon cells of the hematopoietic system. |
| 3 | CXCL8 | C-X-C motif chemokine ligand 8 | IL-8 is a chemotactic factor that attracts neutrophils, basophils, and T-cells, but not monocytes. It is also involved in neutrophil activation. It is released from several cell types in response to an inflammatory stimulus. IL-8(6-77) has a 5-10-fold higher activity on neutrophil activation, IL-8(5-77) has increased activity on neutrophil activation and IL-8(7-77) has a higher affinity to receptors CXCR1 and CXCR2 as compared to IL-8(1-77), respectively. |
| 4 | MMP9 | Matrix metalloproteinase-9 | Proteins of the matrix metalloproteinase (MMP) family are involved in the breakdown of extracellular matrix in normal physiological processes, such as embryonic development, reproduction, and tissue remodeling, as well as in disease processes, such as arthritis and metastasis. |
| 5 | CCL4 | C-C motif chemokine ligand 4 | The protein encoded by this gene is a mitogen-inducible monokine and is one of the major HIV-suppressive factors produced by CD8+ T-cells. The encoded protein is secreted and has chemokinetic and inflammatory functions. |
| 6 | CXCL10 | C-X-C motif chemokine ligand 10 | This antimicrobial gene encodes a chemokine of the CXC subfamily and ligand for the receptor CXCR3. Binding of this protein to CXCR3 results in pleiotropic effects, including stimulation of monocytes, natural killer and T-cell migration, and modulation of adhesion molecule expression. This gene may also be a key regulator of the 'cytokine storm' immune response to SARS-CoV-2 infection. |
| 7 | CD163 | CD163 Molecule | The protein encoded by this gene is a member of the scavenger receptor cysteine-rich (SRCR) superfamily, and is exclusively expressed in monocytes and macrophages. It functions as an acute phase-regulated receptor involved in the clearance and endocytosis of hemoglobin/haptoglobin complexes by macrophages, and may thereby protect tissues from free hemoglobin-mediated oxidative damage. |
| 8 | CCR5 | C-C Motif Chemokine Receptor 5 | This gene encodes a member of the beta chemokine receptor family, which is predicted to be a seven transmembrane protein similar to G protein-coupled receptors. This protein is expressed by T cells and macrophages, and is known to be an important co-receptor for macrophage-tropic virus, including HIV, to enter host cells |
| 9 | CXCR4 | C-X-C Motif Chemokine Receptor 4 | This gene encodes a CXC chemokine receptor specific for stromal cell-derived factor-1. It acts with the CD4 protein to support HIV entry into cells and is also highly expressed in breast cancer cells. |
| 10 | TLR8 | Toll like receptor 8 | The protein encoded by this gene is a member of the Toll-like receptor (TLR) family which plays a fundamental role in pathogen recognition and activation of innate immunity. |
| 11 | CXCL9 | C-X-C motif chemokine ligand 9 | This antimicrobial gene is part of a chemokine superfamily that encodes secreted proteins involved in immunoregulatory and inflammatory processes. The protein encoded is thought to be involved in T cell trafficking. The encoded protein binds to C-X-C motif chemokine 3 and is a chemoattractant for lymphocytes but not for neutrophils. |

**Supplementary Table 2** Transcriptional factors (TFs) of the hub genes.

| **TFs** | **Description** | **P-value** | **Genes** |
| --- | --- | --- | --- |
| RELA | v-rel reticuloendotheliosis viral oncogene homolog A (avian) | 2.81E-11 | CCL4, CXCL10, MMP9, CXCL8, IL1B, CCR5, CXCR4 |
| NFKB1 | nuclear factor of kappa light polypeptide gene enhancer in B-cells 1 | 2.95E-11 | CXCR4, CCR5, IL1B, CXCL8, CXCL10, MMP9, CCL4 |
| NFKBIA | nuclear factor of kappa light polypeptide gene enhancer in B-cells inhibitor, alpha | 1.03E-07 | IL1B, CXCL8, MMP9 |
| ETS2 | v-ets erythroblastosis virus E26 oncogene homolog 2 (avian) | 5.27E-07 | CXCL8, CD163, MMP9 |
| ELF4 | E74-like factor 4 (ets domain transcription factor) | 2.53E-06 | MMP9, CXCL8 |
| IKBKB | inhibitor of kappa light polypeptide gene enhancer in B-cells, kinase beta | 3.79E-06 | CXCL8, MMP9 |
| STAT1 | signal transducer and activator of transcription 1, 91kDa | 9.98E-06 | MMP9, IL1B, CXCL10 |
| YY1 | YY1 transcription factor | 1.27E-05 | IL1B, CCR5, CXCR4 |
| KLF2 | Kruppel-like factor 2 (lung) | 1.39E-05 | CXCR4, CCR5 |
| KLF5 | Kruppel-like factor 5 (intestinal) | 1.97E-05 | MMP9, CXCR4 |
| SNAI2 | snail homolog 2 (Drosophila) | 2.29E-05 | CXCR4, MMP9 |
| JUN | jun proto-oncogene | 5.56E-05 | IL1B, MMP9, CXCL8 |
| ERG | v-ets erythroblastosis virus E26 oncogene homolog (avian) | 8.81E-05 | CXCR4, CXCL8 |
| SIRT1 | sirtuin 1 | 0.000281 | IL1B, MMP9 |
| IRF1 | interferon regulatory factor 1 | 0.000318 | CXCL10, MMP9 |
| EP300 | E1A binding protein p300 | 0.000383 | MMP9, CXCL8 |
| FOS | FBJ murine osteosarcoma viral oncogene homolog | 0.000397 | MMP9, CXCL8 |
| CEBPB | CCAAT/enhancer binding protein (C/EBP), beta | 0.00044 | CXCL8, IL1B |
| SPI1 | spleen focus forming virus (SFFV) proviral integration oncogene spi1 | 0.00047 | IL1B, CD163 |
| HDAC1 | histone deacetylase 1 | 0.000616 | MMP9, CXCL8 |
| ETS1 | v-ets erythroblastosis virus E26 oncogene homolog 1 (avian) | 0.000762 | CXCR4, MMP9 |
| STAT3 | signal transducer and activator of transcription 3 (acute-phase response factor) | 0.00243 | MMP9, CXCL8 |
| SP1 | Sp1 transcription factor | 0.0246 | MMP9, CD163 |

**Supplementary Figure 1** Datasets processing.

**
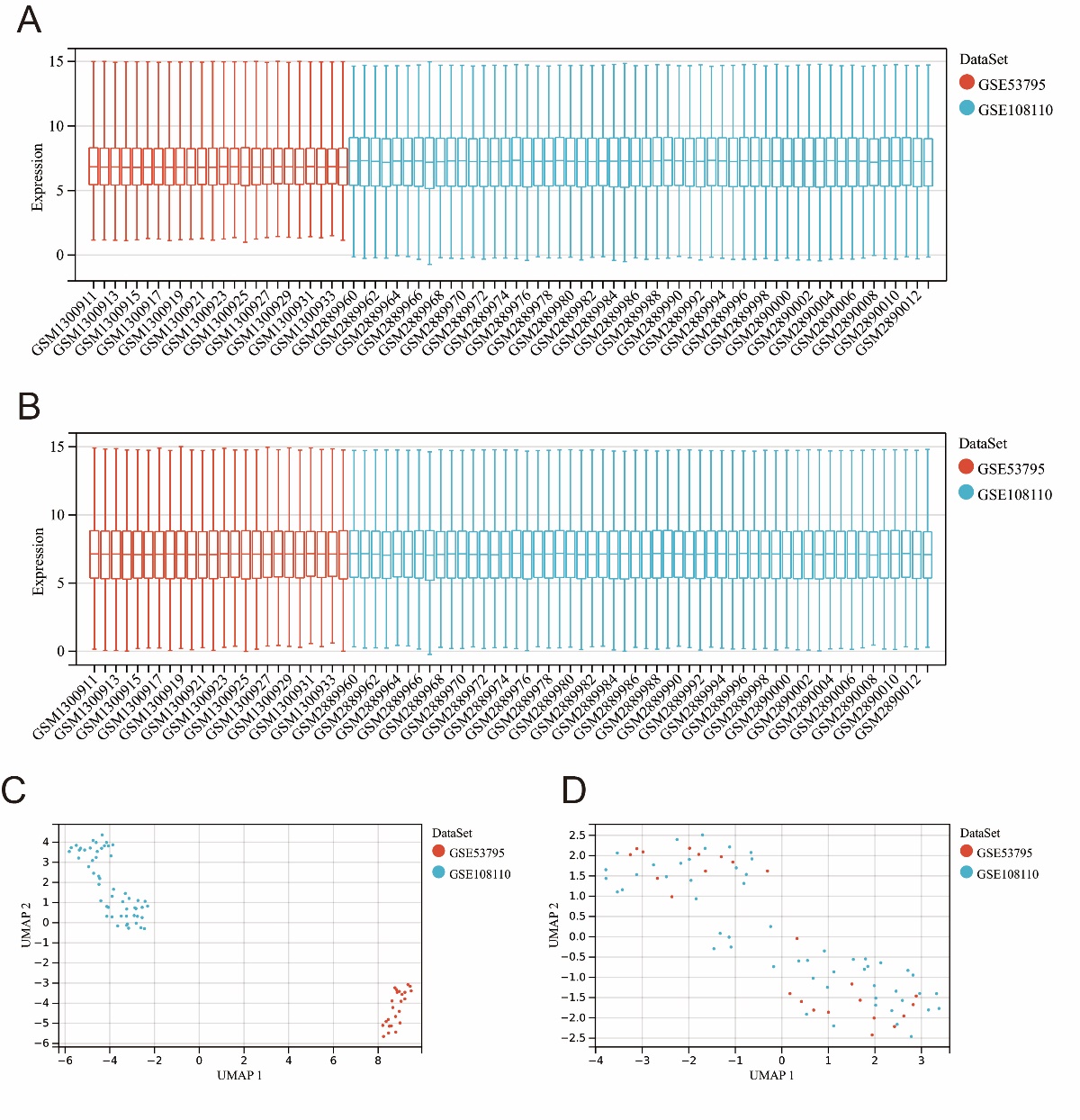
**

Datasets processing. (A) The distributions of the samples in GSE108110 and GSE53795 before the batch effect were eliminated. (B) The distributions of the samples in GSE108110 and GSE53795 after the batch effect were eliminated. (C) UMAP that before the batch effect were eliminated. (D) UMAP that after the batch effect were eliminated. UMAP, Uniform Manifold Approximation and Projection

**Supplementary Figure 2** Enrichment analysis of co-DEGs of rosacea.


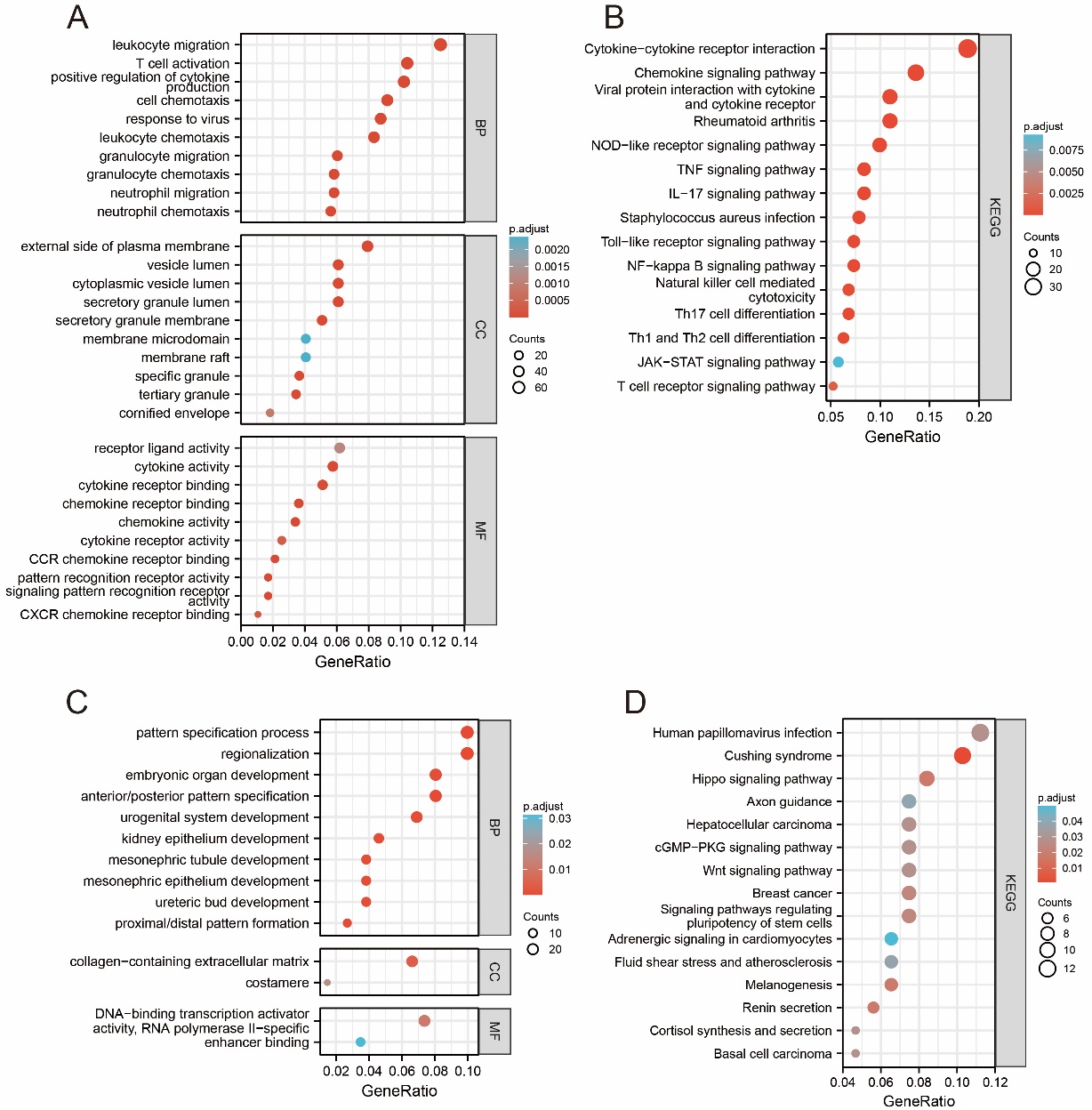


Enrichment analysis of co-DEGs of rosacea. (A, B) GO and KEGG pathway enrichment analysis of the up-regulated co-DEGs of rosacea. (C, D) GO and KEGG pathway enrichment analysis of the down-regulated co-DEGs of rosacea. DEGs, differentially expressed genes; GO, gene ontology; KEGG, Kyoto Encyclopedia of Genes and Genomes.

**Supplementary Figure 3** Enrichment analysis of the DEGs of acne.


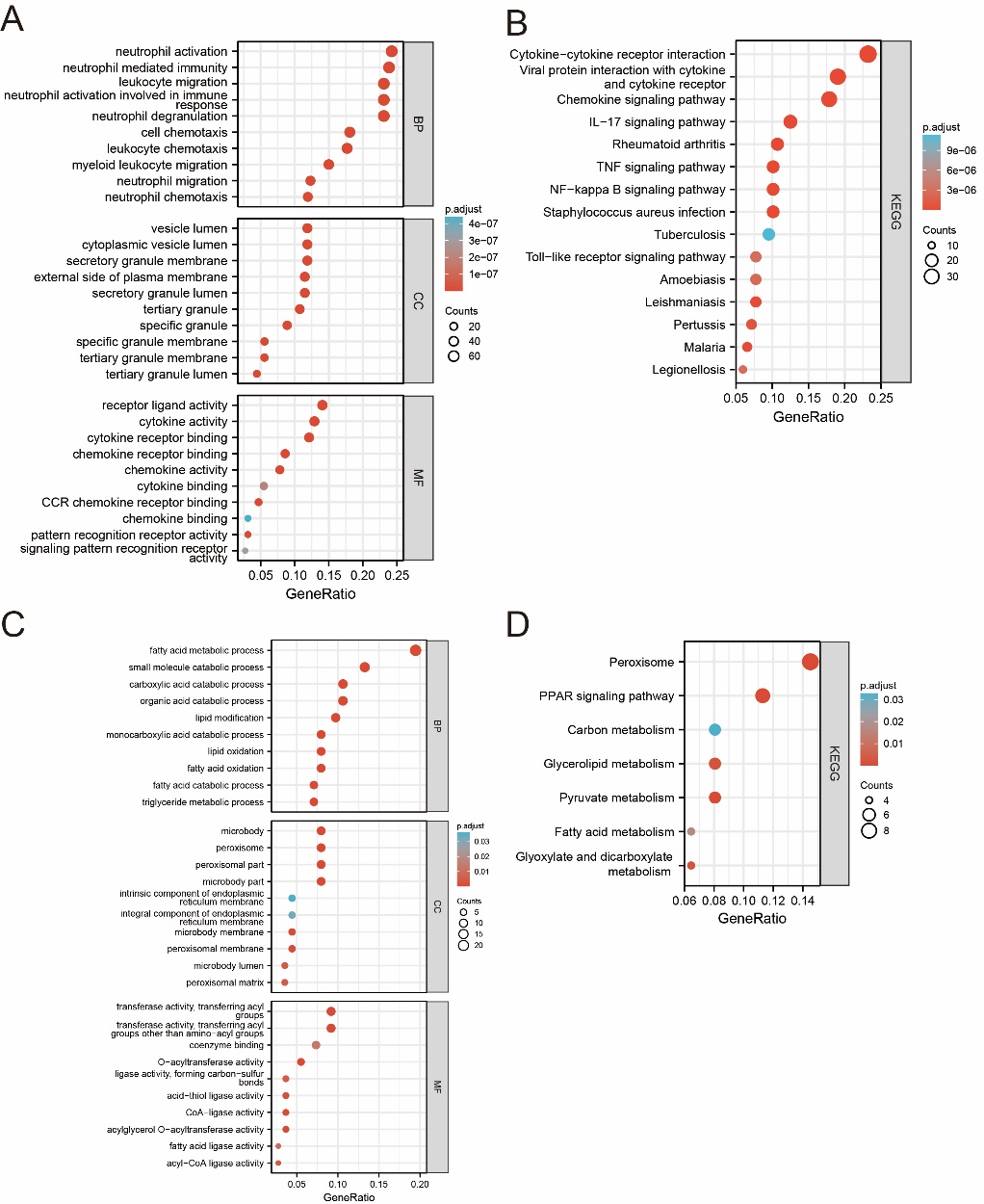


Enrichment analysis of the DEGs of acne. (A, B) GO and KEGG pathway enrichment analysis of the up-regulated DEGs of acne. (C, D) GO and KEGG pathway enrichment analysis of the down-regulated DEGs of acne. DEGs, differentially expressed genes; GO, gene ontology; KEGG, Kyoto Encyclopedia of Genes and Genomes.

**Supplementary Figure 4** The distribution and relative percentage of 22 immune cells.

**
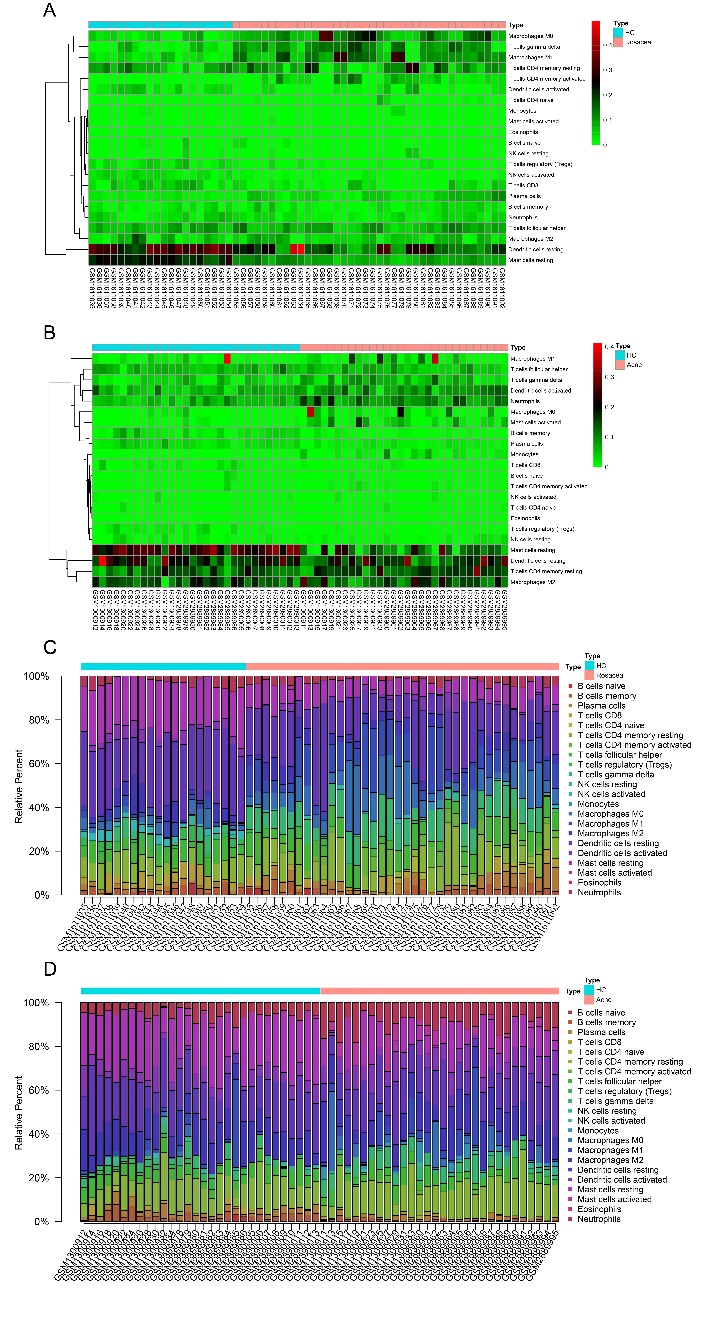
**

The distribution and relative percentage of 22 immune cells. (A) The distribution of 22 immune cells in all samples of rosacea dataset. (B) The distribution of 22 immune cells in all samples of acne dataset. (C) The relative percentage of 22 immune cells in rosacea lesional and HC groups. (D) The relative percentage of 22 immune cells in acne lesional and HC groups. HC, healthy control.

**Supplementary Figure 5** The heatmap of the GSVA scores of the common GSEA pathways.

**
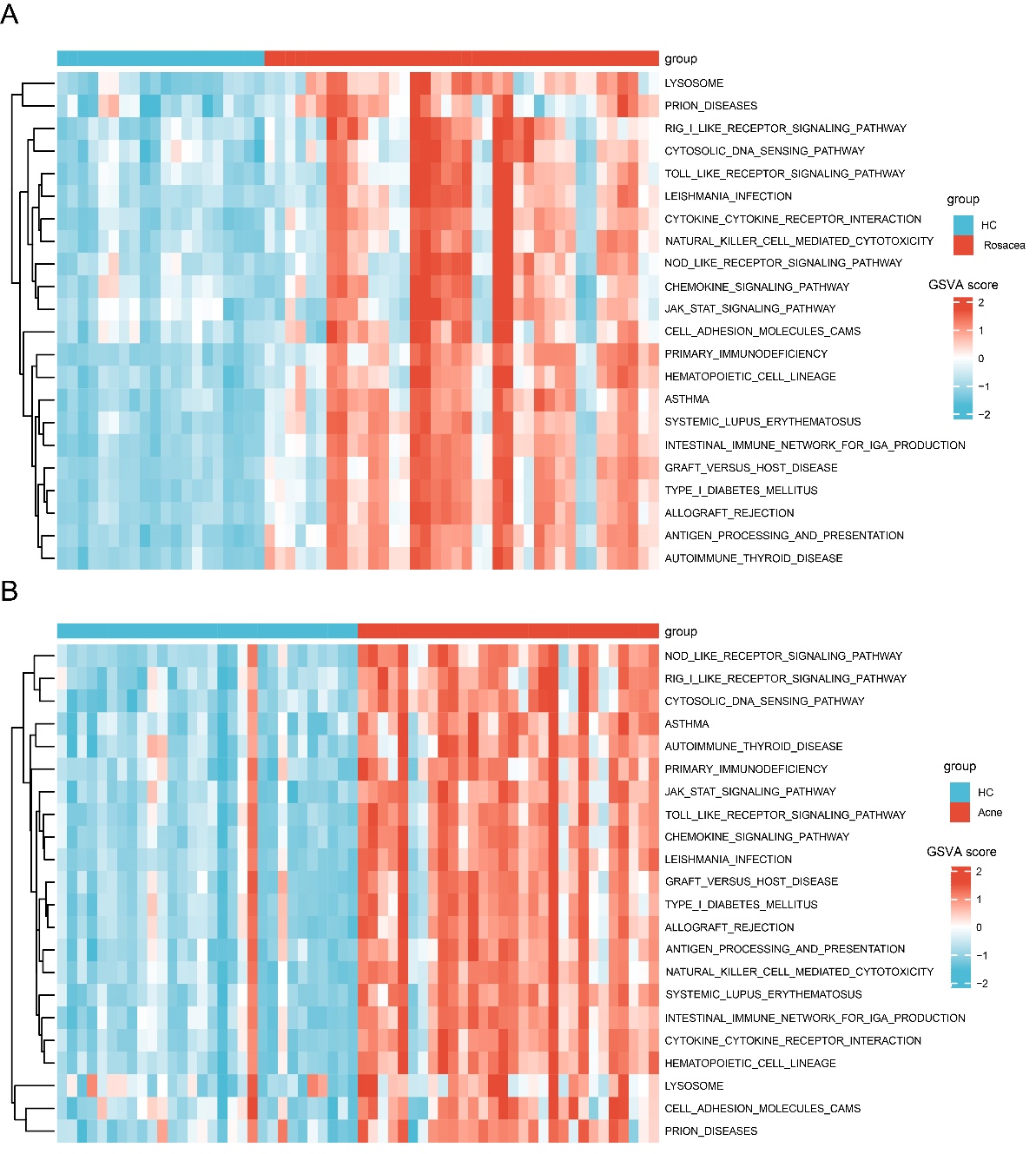
**

The heatmap of the GSVA scores of the common GSEA pathways. (A) GSVA scores of the common GSEA pathways in rosacea dataset. (B) GSVA scores of the common GSEA pathways in acne dataset. GSVA, gene set variation analysis; GSEA, gene set enrichment analysis.

**Supplementary Figure 6** The correlation between 22 immune cells.


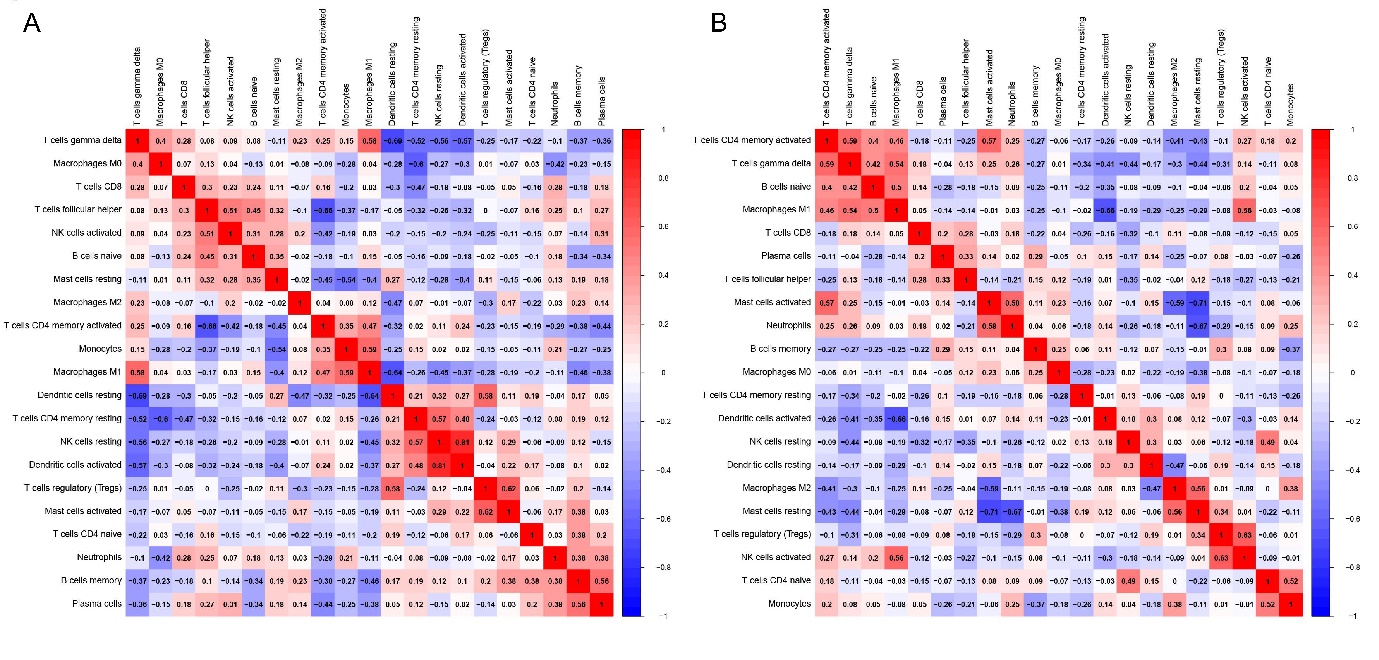


The correlation between 22 immune cells. (A) The correlation between all immune cells in rosacea lesions. (B) The correlation between all immune cells in acne lesions. HC, healthy control.

**Supplementary Figure 7** The lollipop chart of the correlation between hub genes and immune cells in rosacea dataset.

**
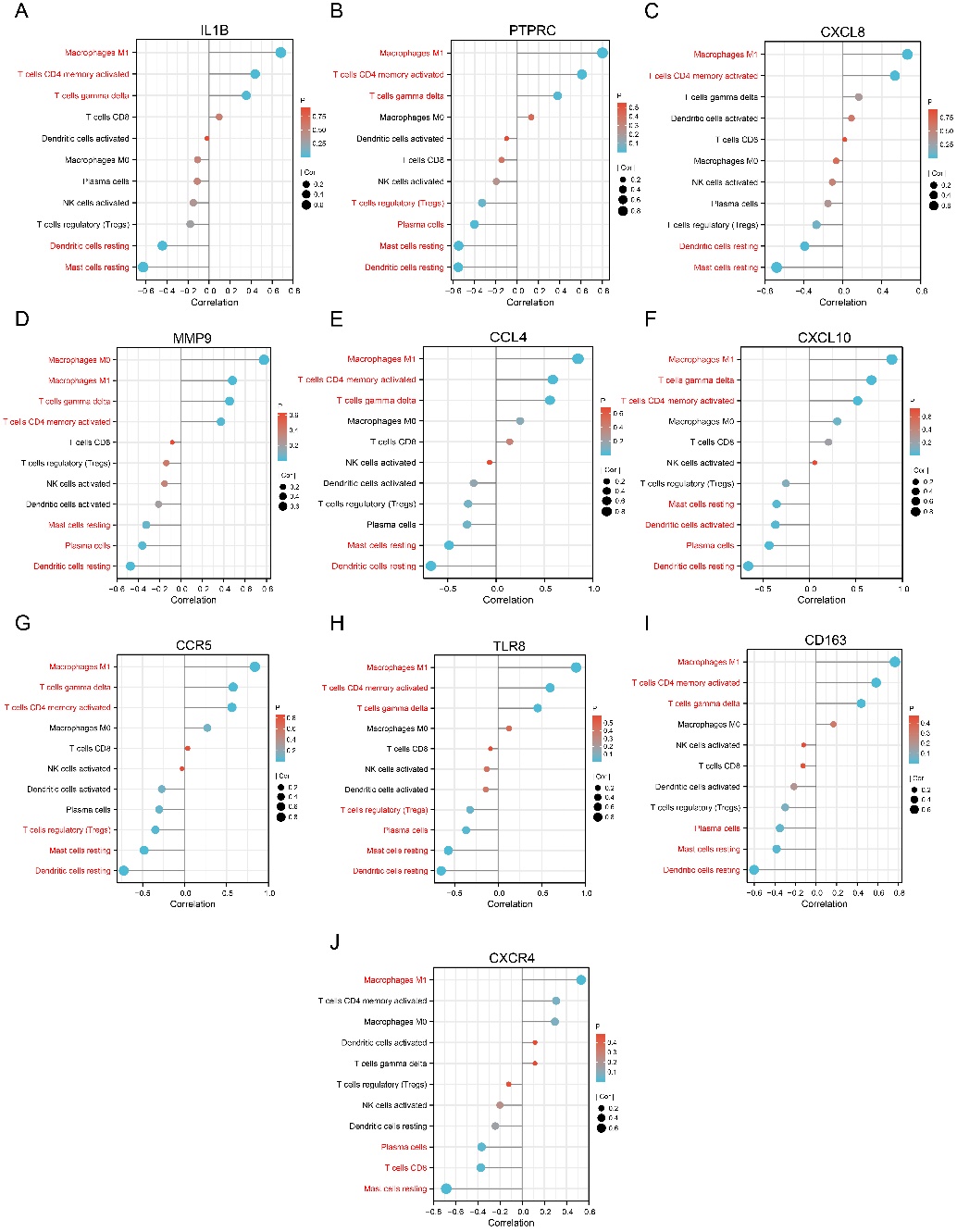
**

The lollipop chart of the correlation between hub genes and immune cells in rosacea dataset. The immune cells which with corresponding p < 0.05 were marked in red.

**Supplementary Figure 8** The lollipop chart of the correlation between hub genes and immune cells in acne dataset.

**
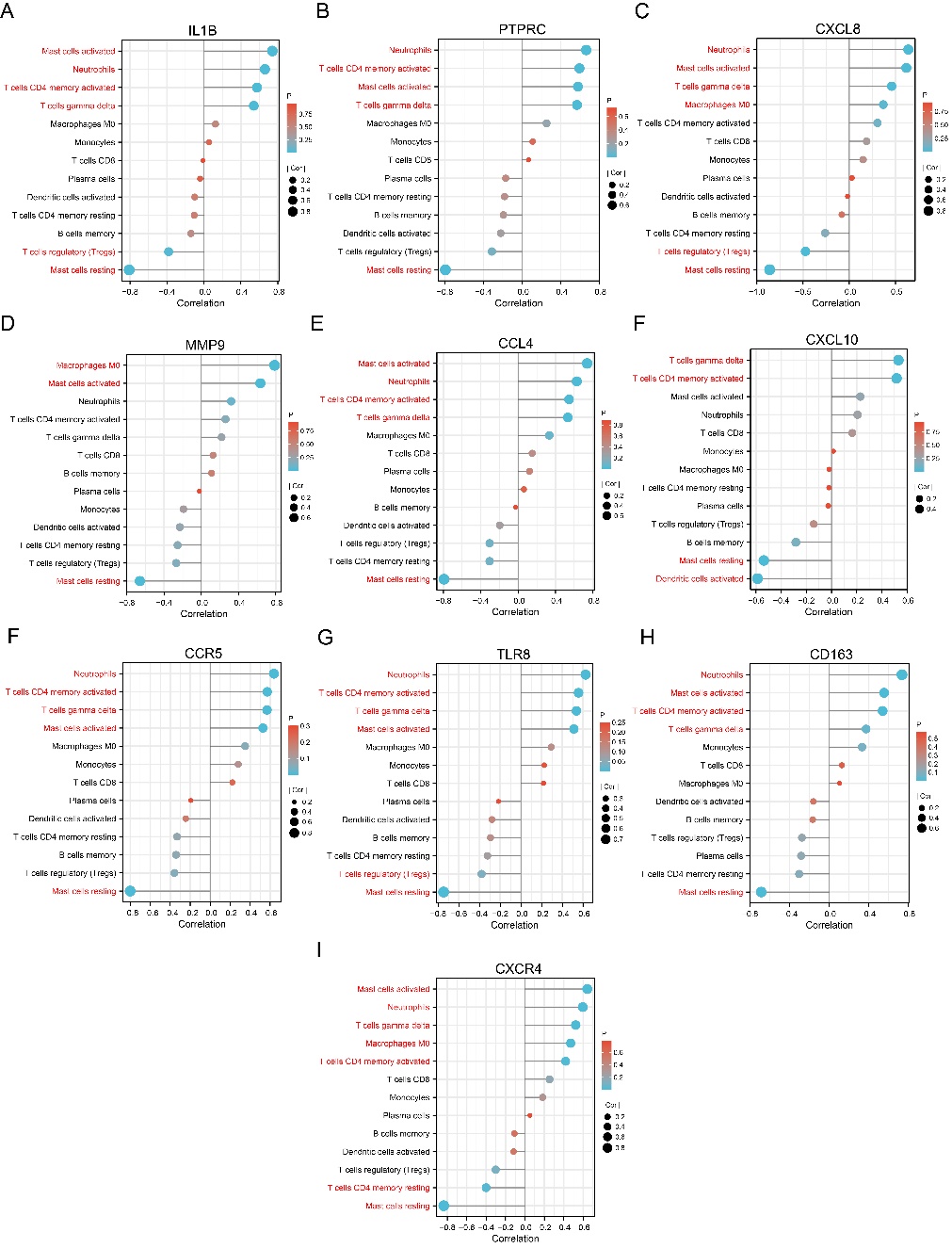
**

The lollipop chart of the correlation between hub genes and immune cells in acne dataset. The immune cells which with corresponding p < 0.05 were marked in red.
